# Differences in meristem size and expression of branching genes are associated with variation in panicle phenotype in wild and domesticated African rice

**DOI:** 10.1186/s13227-017-0065-y

**Published:** 2017-01-28

**Authors:** K. N. Ta, H. Adam, Y. M. Staedler, J. Schönenberger, T. Harrop, J. Tregear, N. V. Do, P. Gantet, A. Ghesquière, S. Jouannic

**Affiliations:** 1 UMR DIADE, IRD, 911, Avenue Agropolis, BP 64501, 34394 Montpellier Cedex 5, France; 2 LMI RICE, IRD, USTH, National Key Laboratory for Plant Cell Biotechnology, Agronomical Genetics Institute, Pham Van Dong Road, Hanoi, Vietnam; 3 UMR DIADE, Université de Montpellier, Place Eugène Bataillon, 34095 Montpellier Cedex 5, France; 4 Department of Botany and Biodiversity Research, University of Vienna, Rennweg 14, Vienna, Austria; 5 Department of Biotechnology-Pharmacology, University of Science and Technology of Hanoi (USTH), 18 Hoang Quoc Viet Road, Hanoi, Vietnam

**Keywords:** Panicle, Tomography, Branching, Meristem fate, African rice

## Abstract

**Background:**

The African rice *Oryza glaberrima* was domesticated from its wild relative *Oryza barthii* about 3000 years ago. During the domestication process, panicle complexity changed from a panicle with low complexity in *O. barthii*, to a highly branched panicle carrying more seeds in *O. glaberrima*. To understand the basis of this differential panicle development between the two species, we conducted morphological and molecular analyses of early panicle development.

**Results:**

Using X-ray tomography, we analyzed the morphological basis of early developmental stages of panicle development. We uncovered evidence for a wider rachis meristem in *O. glaberrima* than in *O. barthii*. At the molecular level, spatial and temporal expression profiles of orthologs of *O. sativa* genes related to meristem activity and meristem fate control were obtained using in situ hybridization and qRT-PCR. Despite highly conserved spatial expression patterns between *O. glaberrima* and *O. barthii*, differences in the expression levels of these early acting genes were detected.

**Conclusion:**

The higher complexity of the *O. glaberrima* panicle compared to that of its wild relative *O. barthii* is associated with a wider rachis meristem and a modification of expression of branching-related genes. Our study indicates that the expression of genes in the *miR156*/*miR529*/*SPL* and *TAW1* pathways, along with that of their target genes, is altered from the unbranched stage of development. This suggests that differences in panicle complexity between the two African rice species result from early alterations to gene expression during reproductive development.

**Electronic supplementary material:**

The online version of this article (doi:10.1186/s13227-017-0065-y) contains supplementary material, which is available to authorized users.

## Background

The African rice *Oryza glaberrima* and the Asian rice *Oryza sativa* are the two species of cultivated rice in the world. While *O. sativa* was domesticated about 10,000 years ago, *O. glaberrima* has a shorter history as it derived from its wild ancestor *Oryza barthii* about 3000 years ago along the Niger River in Mali [1–3]. Recently, different studies provided evidence that African rice domestication was linked with a single domestication origin in West Africa, associated with a severe genetic bottleneck [3–7]. However, in contrast to Asian rice domestication that has been the topic of extensive research, African rice domestication has been less studied in terms of molecular genetics. Although African rice maintains a very low genetic diversity compared to Asian rice [3–6], its close relationship to Asian rice species and its simpler domestication history make it an equally good model to study the evolution of morphological traits and associated gene networks in relation to rice domestication.

Several morphological traits were selected during domestication, including tillering, seed color, seed shattering and many other traits collectively referred to as the “domestication syndrome” [8]. In this context, the inflorescence (i.e., the flower bearing structure) architecture is one of the main morphological traits modified during rice domestications [9]. The architecture of the rice inflorescence (panicle) results from the establishment and activity of apical and axillary meristems derived from the vegetative shoot apical meristem (SAM) [10]. In the reproductive phase, the SAM converts into the rachis meristem (RM), which will give primary branch (PB) meristems until its abortion. These PB meristems will contribute to the establishment of the primary branches as well as axillary meristems, which will contribute to the secondary branch (SB), possibly harboring tertiary branch (TB) meristems. Finally, all the axillary and terminal meristems convert to spikelet (Sp) meristems and then florets [11]. In this way, rice panicle architecture is determined overall by two fundamental phases: the process of meristem establishment and branching; and meristem fate transition from branch/axillary to spikelet meristems. A model of inflorescence evolution was proposed on the basis of differences in the time period required for terminal and axillary meristems to acquire floral fate (i.e., heterochrony) [12]. This model is supported by the analysis of various mutants and detailed transcriptomic time course studies in eudicots [13–15]. In tomato and related nightshades (*Solanaceae*), the diversity of inflorescence structure observed between domesticated and wild relative species is associated with a peak of transcriptome divergence during the reproductive transition, driven by heterochronic shifts [16]. In *O. sativa*, a large number of genes required for the initiation and development of the panicle have been described [17, 18]. Among these genes, two main categories can be defined: (1) the genes related to the branching phase (i.e., establishment and activity of the indeterminate meristems); and (2) the genes related to the transition from indeterminate meristems to spikelet meristems (i.e., spikelet transition) and subsequently the floret phase [11, 18]. The functional analysis of this class of genes led to the hypothesis that panicle complexity in *O. sativa* is governed by the fine-tuning of meristem fate change, through the differential regulation of genes involved in the spikelet transition [11]. However, it was shown recently that the *miR156*/*miR529*/*SPL* regulatory pathway, which plays a key role in the transition from the vegetative to the reproductive phase, is also involved in the control of panicle complexity through the regulation of early acting genes such as *LAX PANICLE1 (LAX1)* and *ABERRANT PANICLE ORGANIZATION2 (APO2)*, as well as the *miR172*/*APETALA2* pathway and the *SEPALLATA*-like gene *PAP2*/*OsMADS34* involved in spikelet and floret development [19]. Within this framework, it is important to determine to what extent these genes might be associated with panicle structure changes associated with rice domestication.

We previously showed that the differential expression of male-gametogenesis-associated, *miR2118*-triggered, 21-nucleotide, phased siRNAs could be associated with the differential rate of spikelet development in panicles between *O. glaberrima* and *O. barthii* [20]. A more thorough investigation is needed to understand the morphological and molecular basis of the observed differential complexity of panicle architecture in the two African rice species. We thus carried out detailed phenotyping of the early developing panicles using high-resolution X-ray tomography [21]. In order to determine whether differences in panicle complexity between the two African rice species might be associated with differential expression of these landmark genes, spatial and temporal expression profiling was performed using a set of *O. sativa* genes likely to play important roles in meristem activity and meristem fate control. Our results revealed that the spatial expression patterns of these genes were conserved. However, differences in their expression levels were observed at a very early stage, reflecting the differential inflorescence meristem size of the two African rice species.

## Results

### African rice panicle structure at mature and early stages

**Fig. 1:**
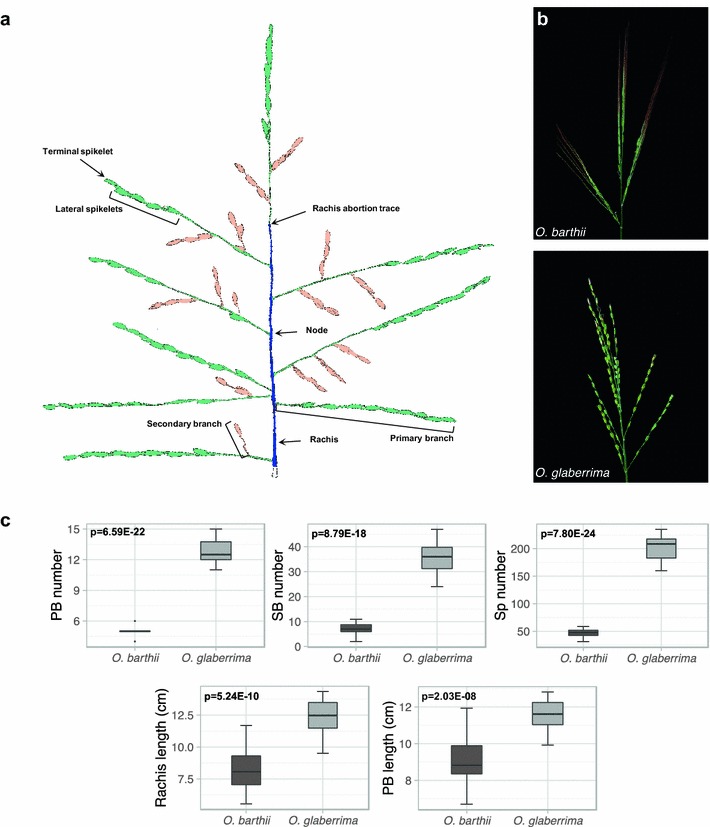
Mature panicle structure in wild and domesticated African species. **a** Spread panicle from *O. glaberrima* (CG14), with the rachis in *blue*, the primary branches in green and the secondary branches in orange. **b** Mature panicle of *O. barthii* (*Ob*) and *O. glaberrima* (*Og*). C. Comparison of panicle traits at the mature stage between *O. barthii* (accession B88) and *O. glaberrima* (accession CG14). *Black lines* within box-plots show the median value; box limits indicate the 25th and 75th percentiles. PB_number, primary branches number; SB_number, secondary branches number; Sp_number, spikelet number. Rachis_length, values of rachis length in cm; PB_length, mean values of the length of primary branches per panicle in cm. Morphological traits were quantified using P-TRAP software [21], *n* = 18 panicles per species. Statistical significance (i.e., *t* test *p* values) between the two species for the different panicle traits is indicated

The rice panicle consists of a series of branches of different orders: rachis (main axis) and higher-order axes (PBs, SBs and sometimes TBs) (Fig. 1a). The single-flowered rice spikelets are established on each panicle branch from apical and lateral meristems. To characterize the phenotype of African rice panicles, we used the P-TRAP software on spread panicles [22], to quantify the main morphological traits in different rice accessions: B88 for *O. barthii* and CG14 for *O. glaberrima* (Fig. 1). There are structural differences in panicle architecture between the two African species (Fig. 1b). Rachis and primary branch lengths are highly variable between panicles of the same accession for both species (Fig. 1c, Additional file 1). For both parameters, *O. glaberrima* panicles display higher mean values than panicles of *O. barthii* (Fig. 1c, Additional file 1). Mature panicles from *O. barthii* (accession B88) possess only a few PBs (mean of 5 ± 1.59 SD) and SBs (7.06 ± 2.31 SD) bearing relatively few spikelets (47.39 ± 7.41 SD) (Fig. 1c, Additional file 1). *O. glaberrima* (accession CG14) mature panicles are more highly branched (i.e., they display higher number of PBs and SBs, 12.67 ± 1.33 SD and 35.50 ± 6.95 SD, respectively), carrying larger numbers of spikelets (199.11 ± 23.97 SD) compared to the wild relative (Fig. 1c, Additional file 1).

**Fig. 2:**
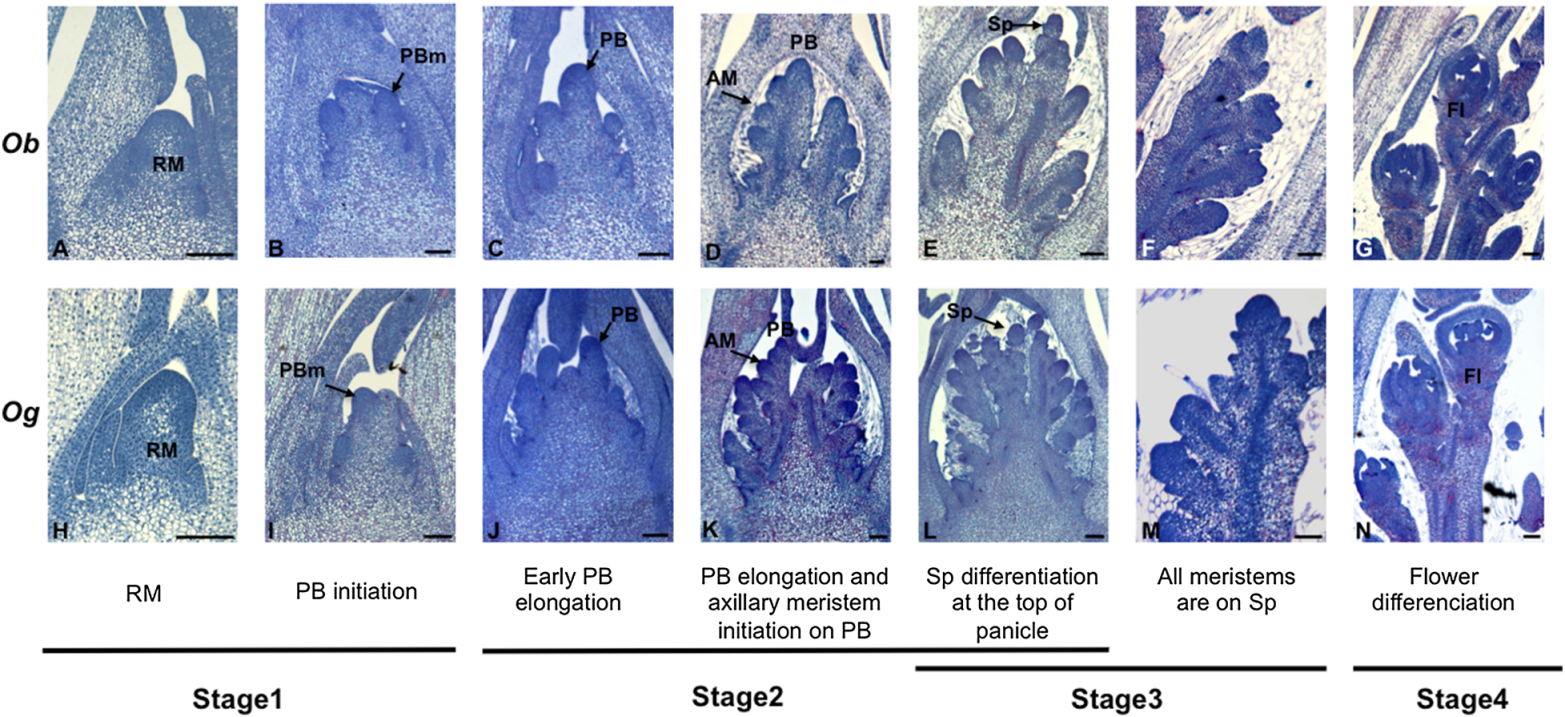
Histological description of early differentiation of African rice panicle and corresponding stages for expression analysis. *O. barthii*: **a**–**g** *O. glaberrima*: **h**–**n**. Developmental stages selected for in situ hybridization and qRT-PCR analyses are indicated in lower panel. Stage 1: unbranched stage with elongation of rachis meristem and formation of primary branch meristems; stage 2: early branching stage with at the end the initiation of spikelet meristem differentiation; stage 3: late branching stage with elongated secondary branch and complete spikelet meristem differentiation; stage 4: floret organ differentiation/development. *AM* axillary meristem, *Fl* flower, *RM* rachis meristem, *PBm* primary branch meristem, *PB* primary branch, *Sp* spikelet. *Scale bars* 100 µm

In this study, we focused on the branching process in terms of meristem establishment and activity and the transition phase to spikelets (i.e., meristem fate), because the branching complexity of the rice panicle is determined at this stage. A description of this developmental framework was carried out in the two African species using classical histology (Fig. 2) and X-ray tomography analyses (Additional files 2 and 3). X-ray tomography allows the acquisition of stacks of high-resolution images from whole-mount material. Subsequently, 3D views can be reconstructed and measurements of morphological structures can be taken independently of sample orientation ([21]; see Additional files 2 and 4). For our analysis, we divided early panicle development into four stages (Fig. 2). Stage 1 corresponds to the elongation of the rachis meristem (RM) and the establishment of primary branch meristem (PBm). In stage 2, the PB elongates and higher-order branches are determined (SBs and TBs) from the initiation of axillary meristems (AMs). The transition from axillary meristems to spikelet meristems (SpMs) occurs at stage 3, and the differentiation of floral organs/flower development occurs at stage 4 (Fig. 2). Using X-ray computed tomography and classical histology, we observed that overall panicle morphology is similar between the two species at the early stages of development. However, during the elongation of the PBs (stage 2), more axillary meristems are initiated along the PBs of *O. glaberrima* compared to those of its wild relative (Fig. 2c–e, j–l). Spikelet differentiation occurs first at the apex of the panicle in both species, after which it progresses toward the base (Fig. 2e, f, l, m; stage 3). At this stage, more SpMs appear to be differentiated in *O. barthii* than in *O. glaberrima*. Spikelet and floral organ differentiation then occurs quickly along the panicle, in an acropetal direction, in both species (Fig. 2f, g, m and n; stages 3 and 4).

**Fig. 3:**
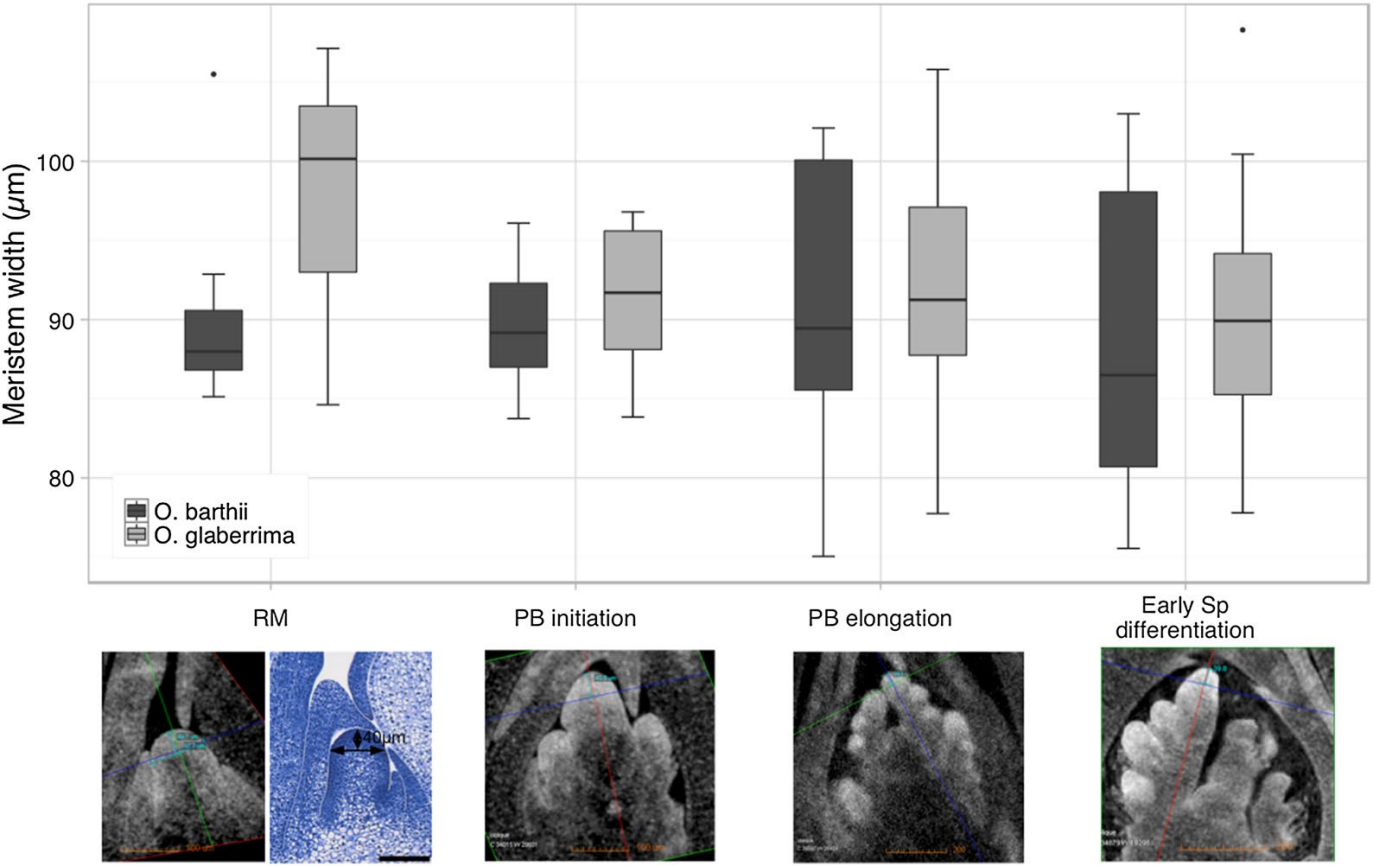
Morphometric traits of panicle meristems in *O. barthii* (*Ob*) and *O. glaberrima* (*Og*). Measurements were taken at different stages of panicle development via virtual sectioning of 3D micro-computed tomography (micro-CT) images: rachis meristem (RM), early primary branch elongation (PB initiation), primary branch elongation before spikelet differentiation (PB elongation) and just after spikelet differentiation at the *top* of the panicle (early spikelet differentiation). *Upper panel*: *box-plots* of meristem width measured from X-ray or histological images (Additional files 2 and 3). *Black lines* within box-plots show the median value; box limits indicate the 25th and 75th percentiles. *Lower panel*: illustration of the considered morphological areas (i.e., width of the meristem at 40 μm from the apical part of the meristem). For the RM stage, meristem widths were quantified from X-ray tomography images (*left*) and classical histological section (*right*) of rachis meristem images (*n* = 9 for each genotype). *Black lines* represent the area of measurement. For the other developmental stages, meristem width was measured for all apical PB meristems of each panicle (n = 9 meristems at least for each genotypes). *Scale bars* = 100 μm. *T* test *p* values: stage RM, *p* = 0.06; stage PB initiation, *p* = 0.22; stage PB elongation, *p* = 0.35; stage Sp, *p* = 0.24

Meristem size (i.e., width at 40 µm from top of median meristem, see Fig. 3, Additional files 2 and 4) was measured at four reliable development stages from X-ray tomography images and histological sections: the RM (stage 1); the early PB elongation (stage 2); late PB elongation during the axillary meristem initiation along the PB (stage 2); and at the stage of Sp differentiation at the top of the panicle (stage 3). For each stage, a minimum of nine meristems was analyzed from at least three independent panicles. Sizes of apical and axillary meristems on PBs at the indeterminate branch stage and at the SpM differentiation stage were observed to be similar between the two species. The only difference that could be observed between the two species was in the rachis meristem width, which tends to be wider in *O. glaberrima* than in *O. barthii* (Fig. 3).

### Spatial expression pattern of African rice orthologs of panicle-related genes

To obtain further insights into the mechanisms regulating differential panicle development in the two African rice species, the expression patterns of two sets of genes known to be associated with panicle development in *O. sativa* were analyzed. Firstly, genes controlling the initiation and/or the maintenance of lateral meristems were used as molecular markers of branch meristem activity, such as *Oryza sativa homeobox1* (*OSH1*), a member of the class I KNOX transcription factor family known to be associated with meristematic cell fate control in angiosperms [23]. The transcription factor-encoding genes *LAX PANICLE1* (*LAX1*) and *SQUAMOSA promoter binding protein*-*like14* (*SPL14*) as well as their microRNA regulators in the panicle *miR529* and *miR156* were also considered in this set of markers, on account of their involvement in axillary meristem establishment and outgrowth during *O. sativa* panicle development [24–27]. The second set of genes related to meristem fate control and included the *SEPALLATA*-like gene *LEAFY HULL STERILE1/OsMADS1* (*LHS1*), which promotes the transition from branch meristems to spikelet meristems [28–31], as well as the *LEAFY* ortholog *ABERRANT PANICLE ORGANIZATION2* (*APO2*) and *TAWAWA1 (TAW1)*, encoding a member of the ALOG family of transcription regulators, the latter two genes both reported to act as suppressors of the transition from branch meristems to spikelet meristems [32–35].

**Fig. 4:**
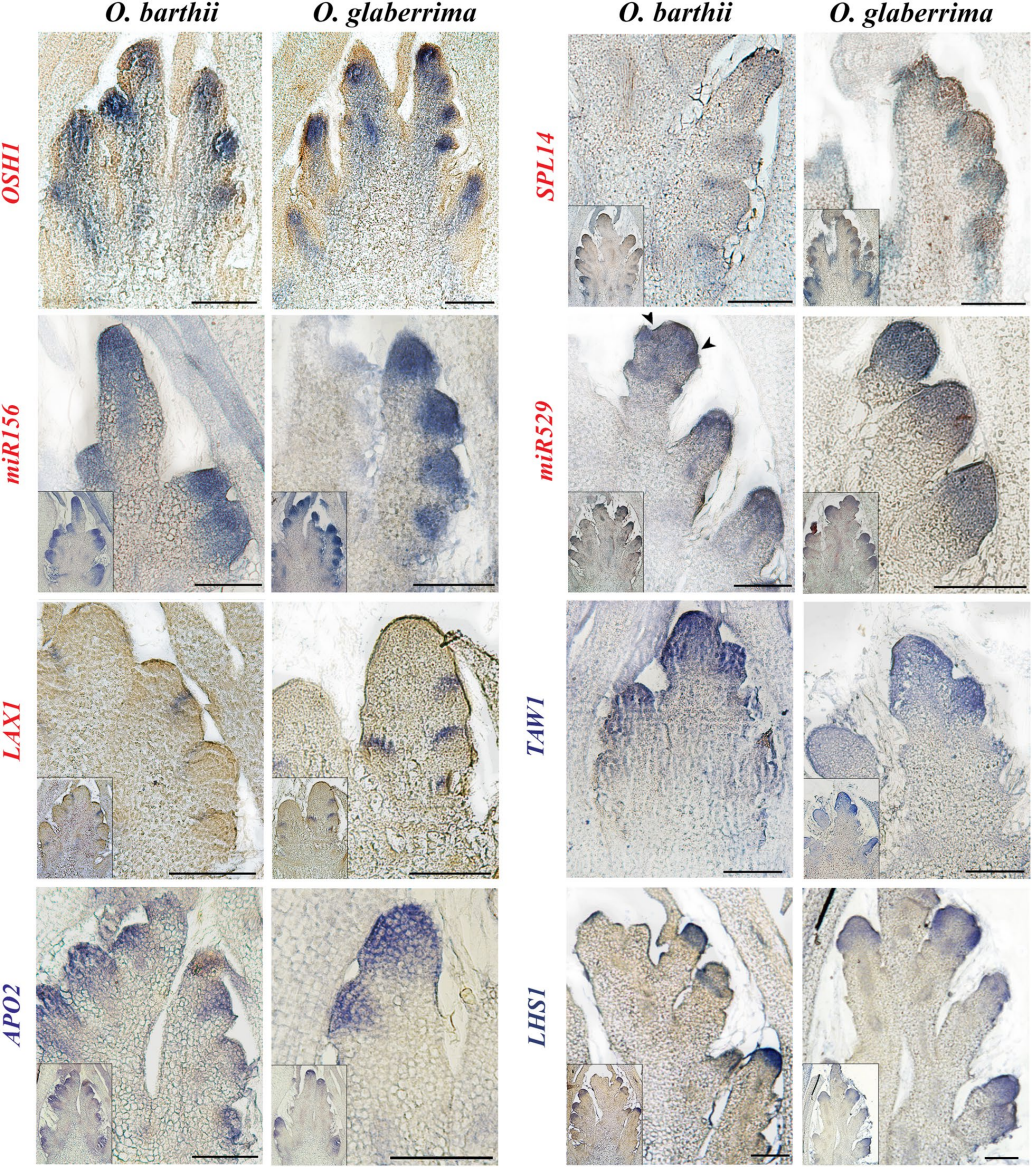
In situ expression patterns of African rice orthologs of panicle-related genes. In situ hybridization analysis of *OSH1*, *SPL14, miR529, miR159, LAX1, APO2, TAW1* and *LHS1* transcripts in branch meristems (stages 2–3) in *O. barthii* and *O. glaberrima.* Genes reported as being involved in branch meristem establishment and outgrowth are indicated in *red*. Those reported as being involved in branch to spikelet fate transition (both activator and inhibitor) are indicated in *blue*. The *inset* photographs show the complete panicle section corresponding to the close-up views of the branch. *Scale bars* 100 µm

Spatial expression profiling of these genes in the two rice species was carried out using in situ hybridization analysis (Fig. 4, Additional file 5). A strong accumulation of *OSH1* transcripts was observed at all developmental stages in the vascular bundles and also in the entire branch meristems (but not in the epidermis) until the stage of initiation of spikelet/floret meristems (Fig. 4, Additional file 5), as previously reported in *O. sativa* [36]. In contrast, *LAX1* transcripts were observed to be restricted specifically to the adaxial boundary region of new BMs and to persist in the young SpMs (Fig. 4, Additional file 5), as reported by [27] in *O. sativa*. Similarly, *SPL14* transcripts were detected in the boundary region of new BMs in both species (Fig. 4, Additional file 5) but not in the spikelets, as reported by [37] in *O. sativa*. In contrast, *miR156* and *miR529*, the *SPL14* microRNA regulators, were detected in the entire new BMs (including epidermis) and persisted in the florets with differentiating organs (Fig. 4, Additional file 5). This finding would suggest that the microRNA-mediated regulation of *SPL14* transcripts acts not as a dampening system but rather as an exclusion/restriction-type mechanism.

*APO2* and *TAW1* ortholog transcripts were detected with a similar patterning: in all branch meristems from early stage 1 of panicle development (Fig. 4, Additional file 5), and also in the SpMs and developing florets. The *APO2* ortholog expression pattern observed in our study is similar to that described by [32] in *O. sativa*, which differs from the pattern described by [34]. In the latter case, a transient down-regulation was observed in late branch meristems and spikelet meristems, which was not observed in our study. The *TAW1* ortholog expression pattern observed here differs from that reported for *O. sativa* [35], in which transcripts were detected only in branch meristems and not in spikelet meristems. In agreement with the report of [38] for *O. sativa*, a strong signal corresponding to *LHS1* transcripts was detected in the spikelet meristem and weakly in the palea and lemma of FMs but not in the inner organs of the differentiating floret nor in branch meristems (Fig. 4, Additional file 5).

Overall, a number of distinct expression patterns were observed for these genes, with profiles that marked branch and/or spikelet meristems or the boundary regions between the axillary meristem and rachis. However, spatial patterning was strictly conserved between the two African rice species and similar to the patterns reported for *O. sativa*.

### Relative expression of panicle-related genes during African rice panicle development

**Fig. 5:**
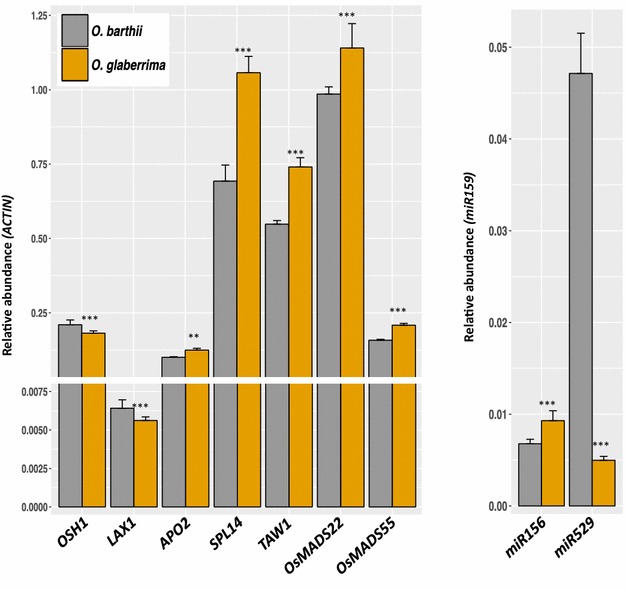
Expression profiling of branching-related genes at unbranched stage in *O. barthii* and *O. glaberrima*. qRT-PCR analysis of *OSH1*, *LAX1*, *SPL14*, *APO2, TAW1*, *OsMADS22*, *OsMADS55*, *miR529* and *miR156* accumulation levels at stage 1 (unbranched stage with elongation of rachis meristem and formation of primary branch meristems) in *O. barthii* (*gray lines*) and in *O. glaberrima* (*orange lines*). Target mRNA and small RNA accumulation levels were normalized using rice *Actin* gene (*LOC_Os03g50885*) transcript and mature *miR159* microRNA accumulation levels, respectively. For mRNAs, the graph is divided into two sections due to scale range. Statistical significances (i.e., *t* test) between the two species for the relative expression levels of each gene are as follows: *p ≤ 0.05; **p ≤ 0.01; ***p ≤ 0.001

In parallel with morphological analysis, relative accumulation of panicle-related gene transcripts in *O. barthii* and *O. glaberrima* was monitored by qRT-PCR at developmental stage 1 (i.e., RM elongation, establishment and initial elongation of PBs; see Fig. 5). Overall, this profiling revealed a higher relative accumulation of transcripts in *O. glaberrima* compared to *O. barthii* for the branching-related genes with one exception, the *LAX1* gene. However, the latter gene displayed a peak of mRNA accumulation at stage 2 in *O. glaberrima* rather than at stage 1 as in *O. barthii* (see Additional file 6). In a same way, a lower accumulation of *OSH1* was observed at stage 1 in *O. glaberrima* but higher accumulation was observed later in development, compared to *O. barthii* (Additional file 6). A lower accumulation of *miR529* in *O. glaberrima* mirrored the higher accumulation of *SPL14* in this species (Fig. 5), in agreement with a *miR529*-dependent regulation of *SPL14* transcript accumulation levels rather than *miR156*-dependency at this stage.

Overall, differences in expression levels were observed for orthologous genes between the two species, with higher expression levels for the branching-related genes *SPL14*, *LAX1*, *APO2*, *TAW1* (and its targets *OsMADS22* and *OsMADS55*) in *O. glaberrima*, leading to the hypothesis that both the *miR156*/*miR529*/*SPL* and *TAW1* regulatory pathways are differentially active between the two African rice species from the unbranched stage of panicle development.

### Divergence of promoter structure between *O. glaberrima* and *O. barthii*

We next investigated whether the differences in expression observed between the two African species might be explained by the genes in question being subject to differential *cis*-regulation. Taking advantage of the recent release of the *O. glaberrima* CG14 genome and *O. barthii* genomic data [7] and local genomic resources, genomic sequences of the landmark genes in CG14 and B88 accessions were analyzed to determine whether any observed polymorphisms fell within gene and promoter sequences. A high conservation of coding sequences was observed between the two African rice species. The recognition sites of the *miR529* and *miR156* microRNAs in the *SPL14* gene of the two African species and *O. sativa* are strictly conserved, indicating that differential expression levels of *SPL14* between the two African rice species were not due to an alteration of these sequences (Additional file 7). In contrast, polymorphic sites between *O. barthii* and *O. glaberrima* were detected in the promoter region of *LAX1* (5 SNPs), *SPL14* (24 SNPs), *APO2* (32 SNPs), *TAW1* (23 SNPs) and *LHS1* (11 SNPs) (Additional file 8). Several GTAC motifs, the binding site of SPL proteins [19], were found in the promoters of *SPL14*, *APO2, LAX1, TAW1* and *LHS1*. However, none of them were affected by polymorphism between the two African rice species. Based on the observed polymorphism in these promoter regions, alteration of expression related to the modification of *cis*-regulatory elements could therefore not be ruled out.

## Discussion

### Spatial expression patterns of panicle-related genes are conserved between the two African rice species

This study was based on the assumption that orthologous genes have a conserved function in African rice species compared to *O. sativa*. This assumption was supported by the fact that there are no non-synonymous changes in orthologous genes between the two species. Moreover, we observed that the spatial expression patterns of the panicle-related genes tested in this study were similar in *O. glaberrima* and *O. barthii*.

The *O. sativa SPL14* and *LAX1* genes and their orthologs in the two African rice species (our study) and in maize (*TASSELSHEATH4* and *BARREN STALK1*, respectively) are expressed at the adaxial boundary adjacent to all branch meristems [25, 37, 39–42]. The *O. sativa miR156*/*miR529*/*SPL14* regulatory pathway was shown to be involved in the control of panicle branching, notably through the regulation of *LAX1* gene expression, which is also reported to be involved in axillary meristem initiation [19, 24, 25, 39–41]. In situ hybridization analysis of *SPL14* gene and miRNA expression patterns in the two African rice species revealed that their expression patterns either did not overlap or only overlapped partially: *miR529* and *miR156* were detected in the center but not in the flank of branch meristems where *SPL14* mRNAs were accumulated (Fig. 5). These spatially separated expression domains suggest a regulatory mechanism based on spatial restriction or mutual exclusion rather than on dampening regulation [43]. In other species, separated patterns were observed for *miR156* and *SPL14*-like genes in *Arabidopsis thaliana* (*SPL9* gene) and in *O. sativa* during the vegetative phase albeit with slight differences, with *SPL14*-like gene transcripts observed along with *miR156* accumulation in both the shoot apical meristem and in leaf primordia [44–46]. This suggests a similar microRNA-dependent regulatory mechanism of *SPL* gene expression, irrespective of the type of microRNA and of the developmental context. However, the spatial expression of *SPL9* was not affected in *A. thaliana se1* and *ago1*-*27* mutants, which have reduced *miR156* accumulation, suggesting that *miR156* is not the main regulator of *SPL9* spatial accumulation in leaf primordia [44].

The *O. sativa APO2*/*RFL* gene, orthologous to the eudicot floral promoting gene *LEAFY* (*LFY*) [14], and the *TAW1* gene belonging to the small *ALOG* (*Arabidopsis LSH1* and *Oryza G1*) gene family have been described as negative regulators of the transition to spikelet meristem fate [32, 34, 35]. However, based on their expression patterns and mutant phenotypes, it would be more accurate to consider these two genes as promoting factors of indeterminate meristematic activity in grass inflorescences. The delay of spikelet meristem specification in the loss of function mutant background may be considered as a consequence of an alteration of branch meristem functioning. In the present study, transcripts of the African rice species orthologs of *O. sativa APO2/RFL* and *TAW1* were detected in both branch and spikelet meristems. The *APO2* ortholog expression pattern observed in our study is similar to the one described by [32] in *O. sativa*. Similarly, a recent analysis of gene expression profiling of reproductive meristem types in early rice inflorescences by laser microdissection has shown that the *APO2/RFL* gene is expressed in spikelet meristems [47]. The *TAW1* ortholog expression pattern observed in the two African rice species differed from the pattern reported in *O. sativa* [35, 47], in which transcripts were detected only in branch meristems and not in spikelet meristems. However, the expression level of the *O. barthii* and *O. glaberrima TAW1* orthologs is still lower in spikelet than in branch meristems (Additional file 6). This suggests that partial divergence of function might have occurred between *TAW1* orthologs genes in Asian and African rice species.

In contrast to the aforementioned genes, the *O. sativa* spikelet-promoting *LHS1/OsMADS1* gene, along with its African rice orthologs, is only expressed in the spikelet meristems ([20, 38]; this study). The maize *LHS1* orthologs *ZMM8* and *ZMM14* are expressed only in the upper floret, and within floral organs of certain sampled taxa, indicating that these genes are involved in the determinacy of the spikelet meristem and in the distinction of upper florets and lower florets in maize inflorescences [48]. In addition, the wheat *LHS1* ortholog (*WLHS1*) is also expressed slightly differently from rice *LHS1*, the transcript of *WLHS1* accumulating at high levels in floret organs (i.e., lemma, palea, pistil, glume) [49]. The *LHS1*-like *SEPALLATA* (*SEP*) genes have been linked with the origin and diversification of the grass spikelet [50].

Overall, we observed a strict conservation of the spatial expression domains of these genes between *O. glaberrima* and *O. barthii*, indicating that the spatial regulation of these genes was not affected during African rice domestication. In addition, an extension of the expression domain of *TAW1* orthologs to spikelets and floret meristems was observed compared to *O. sativa*, suggesting an extension of the functional domain of *TAW1* orthologs in African rice.

### Differential levels of branching-related gene expression at the unbranched developmental stage of African rice panicles

A previous study, based on deep sequencing of panicle-derived small RNA transcriptomes in *O. glaberrima* and *O. barthii*, suggested that the spikelet/floret fate acquisition rate differs between the two species: for a similar morphological complexity at the early branching stage, all meristems are converted into spikelets in *O. barthii*, whereas only those of the apical region of the panicle branches are converted in *O. glaberrima,* as suggested by the expression pattern of the *miR2118*-triggered 21-nt phasiRNA pathway (i.e., *miR2118*, *MEL1*, *lncRNAs* and *phasiRNAs*) and the spikelet-associated MADS-box gene *LHS1/OsMADS1* [20].

The present study revealed that in addition to the spikelet/floret marker genes, genes implicated in panicle branching activity were also differentially expressed, in quantitative terms, between the two species, despite their conserved spatial expression patterns, and this from the unbranched stage (i.e., in the rachis meristem before primary branch establishment). All these genes were more highly expressed in the crop species *O. glaberrima*, with the exception of *miR529*, as might be expected from its known function. These modifications of expression suggest a higher branching activity in *O. glaberrima* compared to its wild relative for the initial reproductive stage in the rachis meristem.

This differential expression during panicle development between the two species may be a consequence of genomic evolution affecting *cis*- and/or *trans*-regulatory mechanisms. This is often the case for traits associated with dynamic processes which are more readily modified through their regulation (i.e., *cis*- and/or *trans*-elements) rather than through coding mutations [51]. Despite the low sequence divergence between the two African rice species [5–7], several SNPs were evident in the promoter regions of the *O. glaberrima LHS1*, *APO2*, *TAW1*, *SPL14* and *LAX1* genes compared to *O. barthii*. Consequently, the possibility of alteration of expression through the modification of *cis*-regulatory elements could not be ruled out.

The observed global alteration of expression of these genes in *O. glaberrima* with respect to its wild relative would suggest that the expression or activity of one or more very early acting factors in panicle development might be affected. Some parallels might be drawn with the key role of the *miR156*/*miR529*/*SPL* regulatory pathway in the control of panicle development through the branch meristem-promoting genes *LAX1* and *APO2*, and also the *miR172*/*AP2* and *PAP2*/*RCN1* regulatory pathways involved in spikelet transition [19]. However, the relationship between the *APO2* and *miR156*/*miR529*/*SPL* pathways still needs to be clarified with regard to the early stages of the transition to the plant reproductive phase. Nevertheless, gene expression profiling of reproductive meristem types in early rice inflorescences by laser microdissection has shown that the *APO2* gene reaches a peak of expression in elongating primary branch/axillary meristems and decreases in spikelet meristems, after the *SPL14* expression peak in initial primary branch meristems [47]. In the same study, the *TAW1* gene as well as its identified target genes (*SVP*-like MADS-box genes *OsMADS22* and *OsMADS55*) displayed a peak of expression in the rachis meristem and decreased in expression between primary branch and spikelet meristems [47]. The genetic and functional relationship between the *miR156*/*miR529*/*SPL* pathway and *TAW1*/*SVP* pathway is not known. However, the present study would suggest a relationship between the two regulatory pathways, as both are more active in *O. glaberrima* than in *O. barthii*.

### Impact of inflorescence meristem size on panicle architecture

Variations in inflorescence architecture can be explained in part by the rate of initiation of branch meristems and by the rate of transition to floral meristems [12, 15]. However, shoot and inflorescence architectures can be initially influenced by meristem size and maintenance [52]. For example, mutations in *CLV* and *ERECTA* pathway genes cause meristems to enlarge, leading to increased shoot and inflorescence branching, more flowers and extra organs in flowers and fruits [53–55]. A comparison of several varieties of *O. sativa* showed that the number of primary branches was related to the initial size of the reproductive apex and that the number of spikelets per primary branch was positively associated with cell division activity during apex growth [56]. Similarly, it was reported that the alteration of *O. sativa* panicle architecture in *apo1* and *apo2* mutants compared to wild-type plants might be associated with a change in inflorescence meristem size in relation to cell proliferation rate [34, 57]. The small panicle with little primary and secondary branching observed in the aforementioned loss of function mutants is associated with a smaller inflorescence meristem displaying reduced cell proliferation activity. Conversely, *APO1*-over-expressing plants, which are characterized by small, packed but highly branched panicles, have a larger inflorescence meristem [57]. Similarly, plants over-expressing the *SPL14* regulator *miR156*, which are characterized by smaller panicles with low branching complexity, also have a smaller inflorescence meristem [19]. It would be interesting to analyze the *SPL* and *TAW1* mis-expressing plants in this context. Overall, these observations would suggest that the modulation of expression of these early acting genes involved in the *miR156*/*miR529*/*SPL* pathway might influence the cell proliferation activity of the inflorescence/rachis meristem, leading to a modulation of branching activity in *O. sativa*.

The rachis meristem in *O. glaberrima* tends to be larger than in its wild relative *O. barthii.* Given the higher expression levels we observed for the early acting *SPL14*, *APO2* and *LAX1* gene orthologs in *O. glaberrima* compared to *O. barthii*, it appears that the difference in panicle architecture between the two species could be related to very early differences in the regulation of inflorescence meristem functioning. However, none of the genes studied here co-localized with genomic regions under a selective sweep in *O. glaberrima* in comparison with *O. barthii* [7], suggesting that these genes were not directly affected by human selection during African rice domestication. This would suggest that one or more factors upstream of this pathway might have been the target of selection.

## Conclusion

The higher complexity of the *O. glaberrima* panicle compared to its wild relative *O. barthii* is associated with a wider inflorescence meristem and a modification of expression of early acting genes affecting branching activity. Our study suggests that the *miR156*/*miR529*/*SPL* and *TAW1* pathways, in association with early targeted genes (*APO2*, *LAX1*, *OsMADS22* and *OsMADS55*), might be affected at the molecular regulatory level. Even though spatial patterns were observed to be conserved, the expression patterns of these genes were found to be modified quantitatively in the rachis meristem. This alteration of gene expression suggests a higher branching activity in *O. glaberrima* and, as a consequence, delayed spikelet meristem fate acquisition. It will be of great interest to dissect the regulatory mechanisms governing the activity of these genes in rice species in order to understand the initial steps of panicle architecture control and its evolution. Moreover, it will be important to highlight to what extent early morphological events and gene expression were affected in similar ways by the two independent domestication events in Asia and Africa, resulting in the phenotypic convergence of panicle complexity that can now be seen.

## Methods

### Plant materials

Plants of *Oryza glaberrima* cv. CG14 (IRRI IRGC acc. #96717, ADN_ID code 142; [6]) and *Oryza barthii* var. B88 (IRRI IRGC acc. #104141, ADN_ID code 589 W; [6]) were grown in a growth chamber for 7 weeks in long-day conditions (14- to 10-h day/night cycle at 32/28 °C and humidity at 60%) at IRD, Montpellier (France). Plants were then transferred and maintained in short-day conditions (10- to 14-h day/night cycle at 32/28 °C and humidity at 60%) for flowering induction. Panicles were collected at four different stages for histology, RNA isolation and in situ hybridization (Fig. 2): stage 1, rachis and primary branch meristem; stage 2, elongated primary branch and secondary meristem, early spikelet differentiation; stage 3, spikelet differentiation; stage 4, young flowers with differentiated organs.

### Histological analyses

All samples were fixed in 4% (w/v) PFA (paraformaldehyde) − 1 × PBS (phosphate-buffered saline) solutions under vacuum for 15 min and then incubated for 16 h at 4 °C. Samples were then treated in serial solutions of 1 × PBS and then dehydrated through a graded ethanol series [50, 70, 80, 90 and 100% (v/v)] for 2 h and stored at 4 °C. They were then transferred into absolute butanol for 2 days and then embedded in resin (Technovit resin, Heraeus Kulzer, Wehrheim, Germany) according to the manufacturer’s instructions. Histological sections (4–5 μm) were made using a rotary microtome (Microm HM 355 S). Sections were double-stained with periodic acid-Schiff’s reagent (Sigma-Aldrich, Lyon, France) and naphthol blue black (NBB, Sigma-Aldrich). Slides were then observed with a Leica DMRB microscope in conjunction with an Evolution MP5.0 color Media Cybernetics camera. Images were processed using Photoshop CS6.

### X-ray micro-computed tomography (micro-CT) and morphometric analysis

For X-ray micro-CT, panicle of the main tiller of three independent plants was collected from 6 to 20 days after floral transition. All samples were treated with a solution of 1% (w/v) of phosphotungstic acid in FAA following the protocols given in [21]. All micro-CT data acquisition was performed on a MicroXCT-200 system (Zeiss Microscopy). Treated samples and scanning conditions are summarized in Additional file 2. 3D data reconstruction was performed via XMReconstructor 8.1.6599 (Zeiss Microscopy). The TMX3D Viewer software (Zeiss Microscopy) was used to perform the morphological and morphometric analyses. In order to carry out exact measurements of meristem size, 2D pictures were generated in TMX3D Viewer software. For each stage, a minimum of nine meristems was analyzed from at least three independent panicles.

### qRT-PCR expression analysis

Total RNAs (mRNAs and small RNAs) from different stages (stage 1 to stage 4) of rice panicle development were extracted using an RNeasy Plant Mini Kit with RLT and RWT buffers (Qiagen, France). DNAse treatments were performed using the RNAeasy-free DNase set (Qiagen). Reverse transcript (RT) samples preparation and qRT-PCR analysis on mRNAs and miRNAs were performed according to [20], using LightCycler 480 thermocycler (Roche, France). Each set of experiments was repeated twice (with three technical replicates each) using independent RTs from the same biological samples. The efficiency of each primer pair used was measured using a dilution series of a mix of RTs from the two accessions and the four stages. The relative quantification method with an efficiency-corrected calculation model [58] was used to evaluate quantitative variation. In miRNA qRT-PCR, accumulation levels were normalized with respect to the mature *miR159* expression level. In mRNA qRT-PCR, mRNAs were normalized with respect to the rice *Actin* gene (*LOC_Os03g50885.1*). The primers used are listed in Additional file 9.

### In situ hybridization

PCR amplifications were performed with gene-specific antisense primers tailed with a T7 RNA polymerase binding site (see Additional file 9 for primer sequences). The resulting DNA fragments were used directly as templates for synthesizing antisense ribo-probes incorporating UTP–digoxigenin (Roche) as the label with the aid of a T7 Maxi Script kit (Ambion). For *miR156* and *miR529* detection, 0.02 M of a 5′ digoxigenin-labeled LNA probe complementary to the target (see Additional file 9 for primer sequences) was used. In situ hybridization experiments were carried out as described by [59]. Detection was performed using the Vector Blue Alkaline Phosphatase Substrate Kit III (Vector Laboratories). Slides were observed and photographed using an Evolution MP5.0 color Media Cybernetics camera in conjunction with a Leica DMRB microscope, and images were processed using Photoshop CS6. Each experiment was repeated at least twice, using at least two sample series (i.e., two paraffin blocks) each time, and the reported spatial patterns were observed in at least two repeats.

### Gene sequencing and data processing

SNP polymorphisms of the genomic sequences of the landmark genes from *O. glaberrima* and *O. barthii* (i.e., promoters, UTRs, CDS and introns) were obtained by using *O. sativa* MSU7.0 reference sequence (http://rice.plantbiology.msu.edu/) for each locus in conjunction with genomic sequences from *O. glaberrima* CG14 [60] and from *O. barthii* B88 accession (kindly provided by F. Sabot, http://irigin.org). The identified polymorphic sites were validated against a previous version of CG14 genomic sequence [7] using the Gramene database (http://blast.gramene.org/Multi/blastview) in conjunction with the *BLASTn* program [61]. A 2.5-Kb-long region upstream from the ATG codon was used for promoter sequence analysis. Identification of putative transcription factor binding sites (TFBSs) in promoter regions was performed using the Genomatix software package (http://www.genomatix.de/) in conjunction with the *O. glaberrima* CG14 genomic sequence from Gramene database.

## Additional files

**Additional file 1.** Mean, median and standard deviation (SD) values of panicle-related traits in *O. barthii* (B88) and *O. glaberrima* (CG14). *PB* primary branch, *Sb* secondary branch, *Sp* spikelet. For each species, *n* = 18.

**Additional file 2.** Illustration of X-ray tomography reconstructed images of the sample scanning and 3D model. **a** Reconstructed transverse section (y–x view) of the sample, **b** reconstructed longitudinal (x–z view) section of the sample, **c** reconstructed longitudinal (y–z view) section of the sample, **d** 3D model of the sample. Meristem width was measured on a reconstructed transverse section at 40 μm from the top of the meristem. Scale bars = 200 μm.

**Additional file 3.** X-ray tomography scanning parameters of *O. glaberrima* and *O. barthii* samples.

**Additional file 4.** longitudinal sections and 3D models of *O. glaberrima* and *O. barthii* panicles at very early developmental stages using X-Ray tomography.

**Additional file 5.** Spatial expression patterns of *OSH1, LAX1, SPL14, miR156, miR529, LHS1, TAW1* and *APO2* genes during panicle development of *O. glaberrima* and *O. barthii*. This figure illustrates the in situ hybridization data during panicle development not presented in Fig. 4 of the main text.

**Additional file 6.** Expression profiling of panicle-related genes during panicle development in *O. barthii* and *O. glaberrima*. qRT-PCR analysis of *OSH1*, *LAX1*, *SPL14*, *APO2, TAW1*, *LHS1*, *OsMADS22*, *OsMADS55*, *miR529* and *miR156* accumulation levels during panicle development (from stage 1 to stage 3) in *O. barthii* (*black lines)* and in *O. glaberrima* (*gray lines*). Target mRNA and small RNA accumulation levels were normalized using the rice *Actin* gene (*LOC_Os03g50885*) transcript and mature *miR159* microRNA accumulation levels, respectively. Expression levels are relative to *O. barthii* B88 stage 1 (y-axis).

**Additional file 7.** *miR156* and *miR529* recognition sites in *SPL14* mRNA target in *O. sativa* (*OsSPL14*), *O. glaberrima* (*OgSPL14*) and *O. barthii* (*ObSPL14*). The single nucleotide change in *O. sativa* from C to A at the *Osa-miR156* targeted site in *OsSPL14* as reported in the *japonica* cultivars Aikawa1 and Shaoniejing (SNJ) by [24, 25], respectively, is highlighted in red. Numbers above the sequence indicate the location of the nucleotide in the *OsSPL14* coding sequence. Dots indicate identical nucleotide sequences in the region corresponding to the recognition sites of *Osa-miR156* and *Osa-miR529*.

**Additional file 8.** Promoter sequence comparisons of *LHS1, APO2* and *SPL14* orthologous genes in *O. glaberrima* and *O. barthii.* Vertical bars represent SNPs in *O. barthii* vs. *O. glaberrima*. Putative transcription factor binding sites related to SNPs are indicated by arrowheads (indicating site orientation). These binding sites are described as being involved in hormone response (ABA and jasmonate, JAS) [62, 63, 65] and in RNA polymerase II transcription activity [64]. The sequence alignments of these sites between *O. barthii* (Ob) and *O. glaberrima* (Og) are indicated on the right, and the corresponding polymorphic site is highlighted.

**Additional file 9.** List of primers used in this study. The underlined sequences correspond to the T7 promoter used for RNA probe synthesis. The sequences in italic correspond to the stem-loop region used for stem-loop qRT-PCRs. Bases shown in square brackets were LNA-modified for in situ hybridization.

## Abbreviations

AM: axillary meristem
Fl: flower
micro-CT: X-ray micro-computed tomography
PB: primary branch
PBm: primary branch meristem
qRT-PCR: quantitative real-time reverse transcription polymerase chain reaction
RM: rachis meristem
SAM: shoot apical meristem
SB: secondary branch
SD: standard deviation
Sp: spikelet
SpM: spikelet meristem
TB: tertiary branch
TFBS: transcription factor binding site
