# Supplementary material for: Differences in meristem size and expression of branching genes are associated with variation in panicle phenotype in wild and domesticated African rice
Source: EvoDevo. 2017 Jan 28;8:2. doi: 10.1186/s13227-017-0065-y (PMC5273837; doi:10.1186/s13227-017-0065-y)
Supplement: Supplementary file 1 — Additional file 1. Mean, median and standard deviation (SD) values of panicle-related traits in O. barthii (B88) and O. glaberrima (CG14). PB primary branch, Sb secondary branch, Sp spikelet. For each species, n = 18. [file 13227_2017_65_MOESM1_ESM.pdf]

Species	Rachis length (cm)		
	Mean	Median	SD
O. barthii	8.28	8.05	1.54
O. glaberrima	12.46	12.47	1.38
	**PB length (cm)**		
	Mean	Median	SD
O. barthii	8.89	8.82	1.36
O. glaberrima	11.61	11.61	0.82
	**PB number**		
	Mean	Median	SD
O. barthii	5.00	8.70	0.59
O. glaberrima	12.67	11.61	1.33
	**SB number**		
	Mean	Median	SD
O. barthii	7.06	36.00	2.31
O. glaberrima	35.50	11.28	6.95
	**Sp number**		
	Mean	Median	SD
O. barthii	47.39	48.00	7.41
O. glaberrima	199.11	208.50	23.97
