# Supplementary figures and images for: Differences in meristem size and expression of branching genes are associated with variation in panicle phenotype in wild and domesticated African rice
Source: EvoDevo. 2017 Jan 28;8:2. doi: 10.1186/s13227-017-0065-y (PMC5273837; doi:10.1186/s13227-017-0065-y)

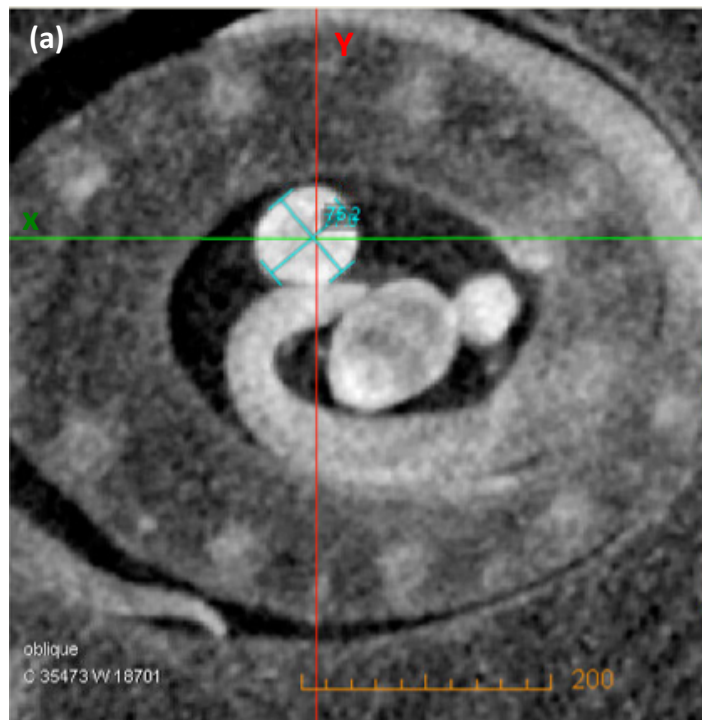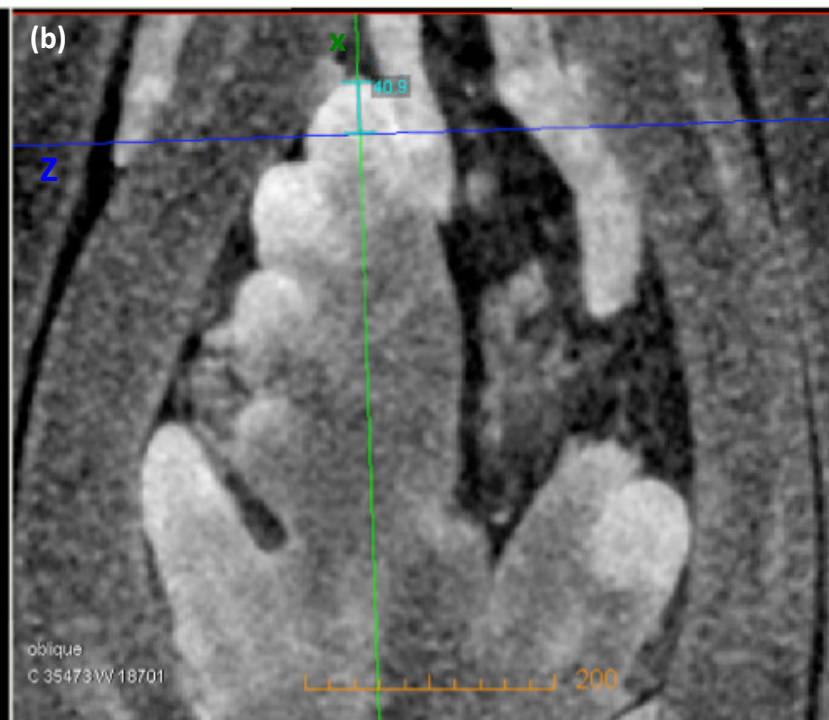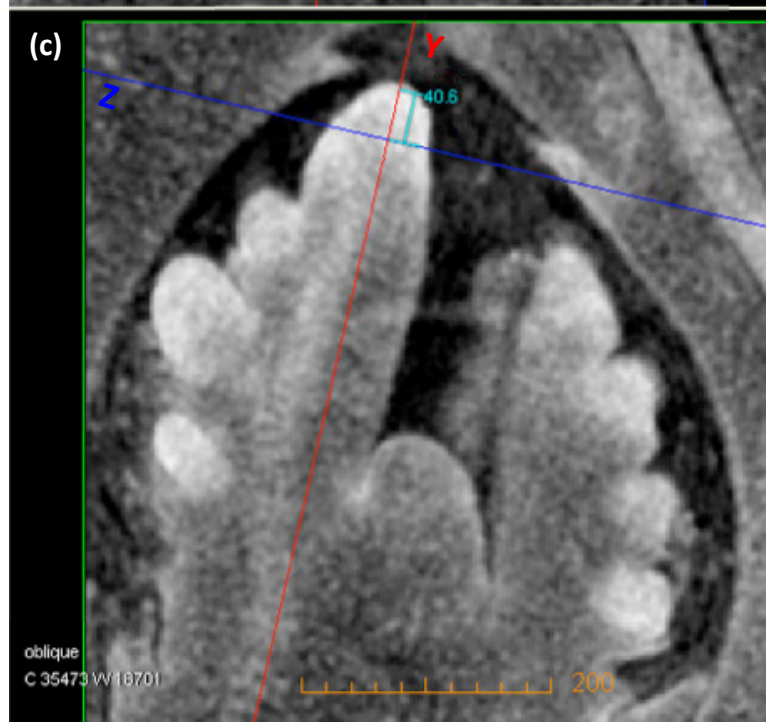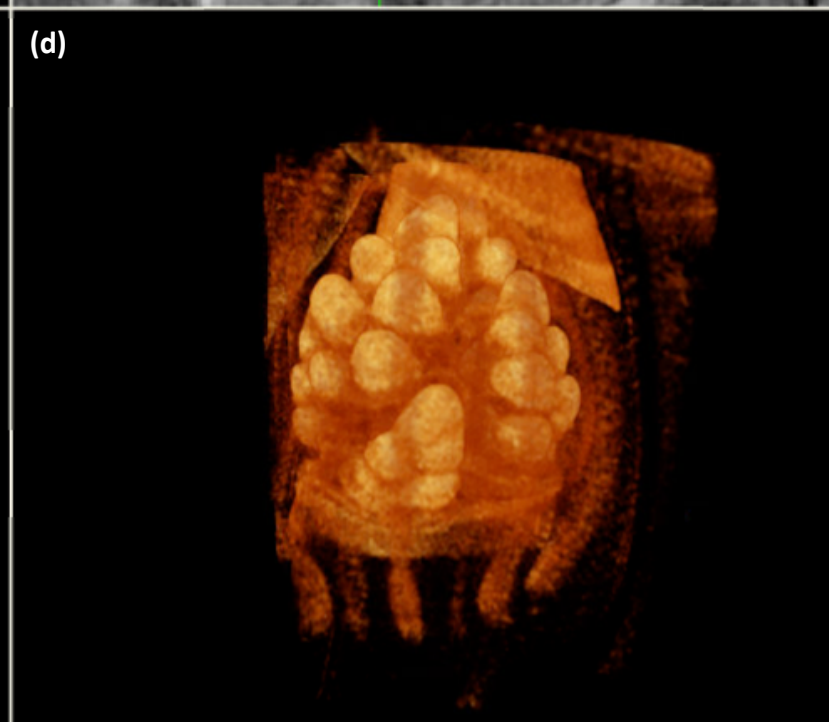

Supplement: Supplementary file 2 — Additional file 2. Illustration of X-ray tomography reconstructed images of the sample scanning and 3D model. a Reconstructed transverse section (y–x view) of the sample, b reconstructed longitudinal (x–z view) section of the sample, c reconstructed longitudinal (y–z view) section of the sample, d 3D model of the sample. Meristem width was measured on a reconstructed transverse section at 40 μm from the top of the meristem. Scale bars = 200 μm. [file 13227_2017_65_MOESM2_ESM.pdf]

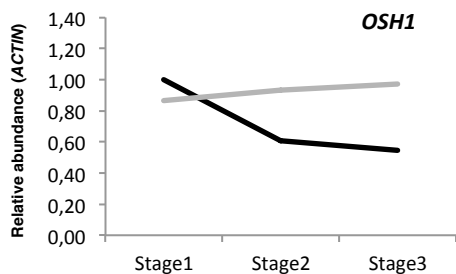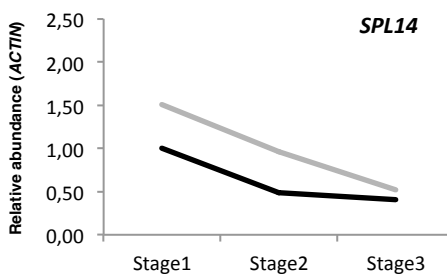

— Ob-B88  
— Og-CG14

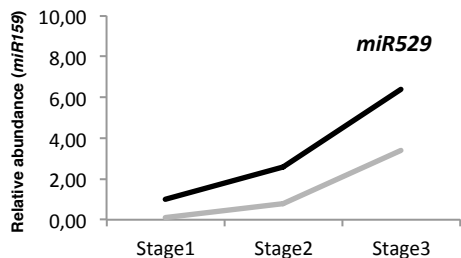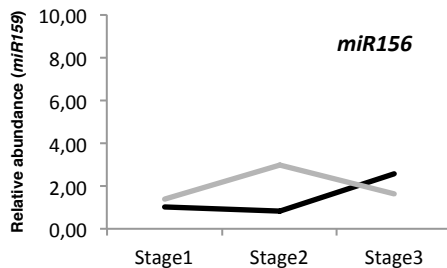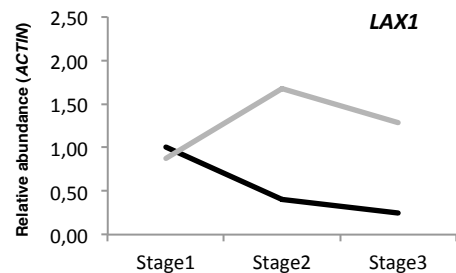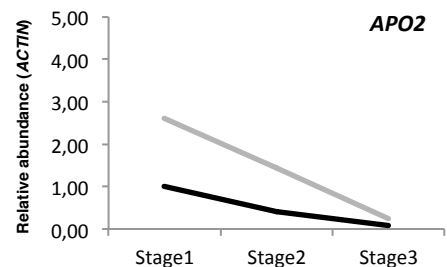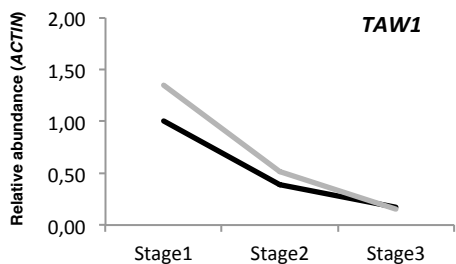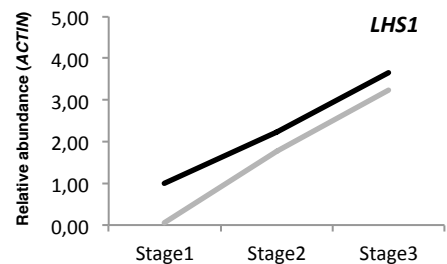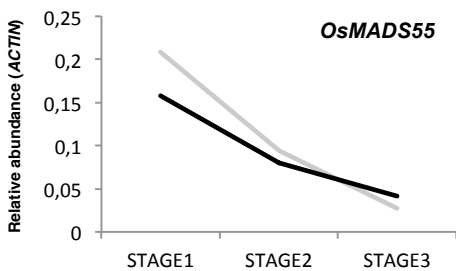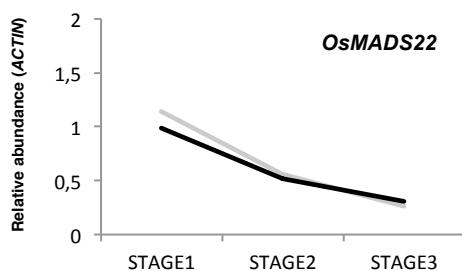

Supplement: Supplementary file 6 — Additional file 6. Expression profiling of panicle-related genes during panicle development in O. barthii and O. glaberrima. qRT-PCR analysis of OSH1, LAX1, SPL14, APO2, TAW1, LHS1, OsMADS22, OsMADS55, miR529 and miR156 accumulation levels during panicle development (from stage 1 to stage 3) in O. barthii (black lines) and in O. glaberrima (gray lines). Target mRNA and small RNA accumulation levels were normalized using the rice Actin gene (LOC_Os03g50885) transcript and mature miR159 microRNA accumulation levels, respectively. Expression levels are relative to O. barthii B88 stage 1 (y-axis). [file 13227_2017_65_MOESM6_ESM.pdf]
