# Supplementary material for: Differences in meristem size and expression of branching genes are associated with variation in panicle phenotype in wild and domesticated African rice
Source: EvoDevo. 2017 Jan 28;8:2. doi: 10.1186/s13227-017-0065-y (PMC5273837; doi:10.1186/s13227-017-0065-y)
Supplement: Supplementary file 3 — Additional file 3. X-ray tomography scanning parameters of O. glaberrima and O. barthii samples. [file 13227_2017_65_MOESM3_ESM.pdf]

Species	Sample name	Acceleration voltage [kV]	Source current [µA]	Objective	Camera binning	Pixel size [µm]	Exposure time [s]	Pictures per sample
Oryza glaberrima	Og1-2	48	19	10x	2	1.92	12	728
Oryza glaberrima	Og2-2	48	19	10x	2	1.9006	12	728
Oryza glaberrima	Og3-2	48	19	10x	2	1.9006	12	728
Oryza glaberrima	Og4-2	45	23	10x	2	1.9205	10	728
Oryza glaberrima	Og6-2	45	23	10x	2	1.9205	10	728
Oryza glaberrima	Og7-2	45	23	10x	2	1.9274	10	728
Oryza glaberrima	Og8-2	45	23	10x	2	1.9341	10	728
Oryza glaberrima	Og3-3	50	24	4x	2	4.6514	10	728
Oryza glaberrima	Og4-3	53	23	10	2	1.8543	12	728
Oryza glaberrima	Og5-3	53	23	4x	2	1.9554	11	728
Oryza glaberrima	Og6-3	56	24	10	2	1.9554	11	728
Oryza glaberrima	Og7-3	56	24	10	2	1.9639	11	728
Oryza glaberrima	Og8-3	56	24	10	2	1.9639	11	728
Oryza glaberrima	Og5-2	45	22	4	2	4.8175	11	728
Oryza glaberrima	Og9-1	55	24	10	2	2.1226	15	728
Oryza glaberrima	Og10-1	55	24	10	2	1.9394	15	728
Oryza glaberrima	Og11-1	55	24	10	2	1.9066	15	728
Oryza glaberrima	Og12-1	55	24	10	2	1.9837	15	728
Oryza glaberrima	Og13-1	55	24	10	2	1.9342	15	728
Oryza glaberrima	Og15-1	55	24	10	2	1.7375	15	728
Oryza glaberrima	Og9-2	60	25	10	2	1.9515	15	728
Oryza glaberrima	Og10-2	60	25	10	2	1.914	15	728
Oryza glaberrima	Og11-2	50	22	4	2	4.8336	11	728
Oryza glaberrima	Og12-2	50	22	4	2	4.3942	11	728
Oryza glaberrima	Og13-2	55	24	10	2	1.914	15	728
Oryza glaberrima	Og15-2_part1	55	24	10	2	1.8647	15	728
Oryza glaberrima	Og15-2_part2	55	24	10	2	1.8647	15	728
Oryza glaberrima	Og10-3	60	12	10	2	1.8373	16	728
Oryza glaberrima	Og11-3	60	12	10	2	1.6702	15	728
Oryza glaberrima	Og13-3	60	12	10	2	1.8373	15	728
Oryza glaberrima	Og15-3_part1	60	12	10	2	1.8373	15	728
Oryza glaberrima	Og15-3_part2	60	12	10	2	1.8373	15	728
Oryza barthii	Ob1-1	48	16	4	2	4.7864	10	728
Oryza barthii	Ob2-1	53	22	10	2	1.9007	13	728
Oryza barthii	Ob3-1	53	22	10	2	1.9174	13	728
Oryza barthii	Ob4-1	53	22	10	2	1.9174	12	728
Oryza barthii	Ob5-1	53	22	10	2	1.9076	12	728
Oryza barthii	Ob6-1	53	22	10	2	1.9473	12	728
Oryza barthii	Ob7-1	53	22	10	2	1.9009	12	728
Oryza barthii	Ob8-1	53	15	10	2	1.9007	13	728
Oryza barthii	Ob1-2	53	15	4	2	4.8338	10	728
Oryza barthii	Ob2-2	53	15	10	2	1.9007	13	728
Oryza barthii	Ob3-2	53	15	10	2	1.9341	13	728
Oryza barthii	Ob4-2	53	15	10	2	1.9206	13	728
Oryza barthii	Ob5-2	53	15	10	2	1.924	13	728
Oryza barthii	Ob6-2	53	15	10	2	1.9005	13	728
Oryza barthii	Ob3-3	53	22	10	2	1.8844	13	728
Oryza barthii	Ob4-3	53	22	10	2	1.9036	13	728
Oryza barthii	Ob5-3	53	22	10	2	1.9036	10	728
Oryza barthii	Ob6-3	50	22	4	2	4.7679	10	728
Oryza barthii	Ob7-3	53	22	10	2	1.9007	13	728
Oryza barthii	Ob8-3	53	22	4	2	4.8014	8	728
Oryza barthii	Ob7-2	53	22	4	2	4.076	10	728
Oryza barthii	Ob9-1	55	10	10	2	1.8938	18	728
Oryza barthii	Ob10-1	55	10	4	2	3.9504	10	728
Oryza barthii	Ob11-1	55	10	10	2	1.9511	18	728
Oryza barthii	Ob12-1	55	10	4	2	4.5707	6	728
Oryza barthii	Ob8-2	55	10	10	2	1.8893	18	728
Oryza barthii	Ob9-3	53	15	10	2	1.9006	15	728
Oryza barthii	Ob10-3	53	15	4	2	4.1656	8	728
Oryza barthii	Ob11-3_part1	53	15	4	2	4.6437	6	728
Oryza barthii	Ob11-3_part2	53	15	4	2	4.6437	6	728
Oryza barthii	Ob12-3	53	15	4	2	3.8803	8	728
Oryza barthii	Ob13-3_part1	53	15	4	2	4.6437	6	728
Oryza barthii	Ob13-3_part2	53	15	4	2	4.6437	6	728
Oryza barthii	Ob13-1_part1	53	15	4	2	4.678	5	728
Oryza barthii	Ob13-1_part2	53	15	4	2	4.678	5	728
Oryza barthii	Ob13-1_part3	53	15	4	2	4.678	5	728
Oryza barthii	Ob14-1_part1	53	15	4	2	4.678	5	728
Oryza barthii	Ob14-1_part2	53	15	4	2	4.678	5	728
Oryza barthii	Ob14-3	53	15	4	2	3.537	7	728
Oryza barthii	Ob15-1	50	17	1	2	19.5659	10	728
Oryza barthii	Ob15-3	40	14	LFOV	2	37.6713	9	1400
Oryza barthii	Ob14-2_part1	45	12	1	2	17.2398	10	728
Oryza barthii	Ob14-2_part2	45	12	1	2	17.2398	10	728
Oryza barthii	Ob9-2	50	18	10	2	1.8868	15	728
Oryza barthii	Ob10-2	50	18	10	2	1.8868	15	728
Oryza barthii	Ob11-2	50	18	10	2	1.9006	15	728
Oryza barthii	Ob12-2	50	18	10	2	1.9006	13	728
Oryza barthii	Ob13-2_part1	45	18	1	2	19.1506	5	728
Oryza barthii	Ob13-2_part2	45	18	1	2	19.1506	5	728
Oryza barthii	Ob15-2_part1	45	18	4	2	4.7321	5	728
Oryza barthii	Ob15-2_part2	45	18	4	2	4.7321	5	728
