# Supplementary material for: Differences in meristem size and expression of branching genes are associated with variation in panicle phenotype in wild and domesticated African rice
Source: EvoDevo. 2017 Jan 28;8:2. doi: 10.1186/s13227-017-0065-y (PMC5273837; doi:10.1186/s13227-017-0065-y)
Supplement: Supplementary file 4 — Additional file 4. longitudinal sections and 3D models of O. glaberrima and O. barthii panicles at very early developmental stages using X-Ray tomography. [file 13227_2017_65_MOESM4_ESM.pdf]

Additional File 4_Movies: longitudinal sections and 3D models of *O. glaberrima* and *O. barthii* panicles at very early developmental stages using X-Ray tomography. The movies are available by downloading using the following web link: https://mega.nz/#F!Q5VEDAKA!BD-XLBgwtAmHLbQqu5kgRg

Movie 1. Longitudinal section series played as a video through the Rachis meristem stage (stage 1) of *O. barthii*. Scale Bar = 100µm.

Movie 2. Animation of a 3D model of a rachis meristem stage (stage 1) of *O. barthii*.

Movie 3. Longitudinal section series played as a video through the Rachis meristem stage of *O. glaberrima*. Scale Bar = 200µm.

Movie 4. Animation of a 3D model of a Rachis meristem stage of *O. glaberrima*.

Movie 5. Longitudinal section series played as a video through a panicle (stage1 PB initiation) of *O. barthii*. Scale Bar = 100µm.

Movie 6. Animation of a 3D model of a panicle (stage 1 PB initiation) of *O. barthii*.

Movie 7. Longitudinal section series played as a video through a panicle (stage 1 PB initiation) of *O. glaberrima*. Scale Bar = 100µm.

Movie 8. Animation of a 3D model of a panicle (stage 1 PB initiation) of *O. glaberrima*.

Movie 9. Longitudinal section series played as a video through a panicle (stage2 PB elongation) of *O. barthii*. Scale Bar = 200µm.

Movie 10. Animation of a 3D model of a panicle (stage 2 PB elongation) of *O. barthii*.

Movie 11. Longitudinal section series played as a video through a panicle (stage1 PB initiation) of *O. glaberrima*. Scale Bar = 100µm.

Movie 12. Animation of a 3D model of a panicle (stage 1 PB elongation) of *O. glaberrima*.

Movie 13. Longitudinal section series played as a video through a panicle (stage2 PB elongation and AM initiation) of *O. barthii*. Scale Bar = 200µm.

Movie 14. Animation of a 3D model of a panicle (stage 2 PB elongation and AM initiation) of *O. barthii*.

Movie 15. Longitudinal section series played as a video through a panicle (stage 2 PB initiation and AM initiation) of *O. glaberrima*. Scale Bar = 500µm.

Movie 16. Animation of a 3D model of a panicle (stage 2 PB elongation and AM initiation) of *O. glaberrima*.

Movie 17. Longitudinal section series played as a video through a panicle (stage 2-3 Sp differentiation) of *O. barthii*. Scale Bar = 200µm.

Movie 18. Animation of a 3D model of a panicle (stage 2-3 Sp differentiation) of *O. barthii*.

Movie 19. Longitudinal section series played as a video through a panicle (stage 2-3 Sp differentiation) of *O. glaberrima*. Scale Bar = 200µm.

Movie 20. Animation of a 3D model of a panicle (stage 2-3 Sp differentiation) of *O. glaberrima*.

Movie 21. Longitudinal section series played as a video through a panicle (stage 3 Sp differentiation) of *O. barthii*. Scale Bar = 200µm.

Movie 22. Animation of a 3D model of a panicle (stage 3 Sp differentiation) of *O. barthii*.

Movie 23. Longitudinal section series played as a video through a panicle (stage 3 Sp differentiation) of *O. glaberrima*. Scale Bar = 200µm.

Movie 24. Animation of a 3D model of a panicle (stage 3 Sp differentiation) of *O. glaberrima*.
