# Supplementary material for: Differences in meristem size and expression of branching genes are associated with variation in panicle phenotype in wild and domesticated African rice
Source: EvoDevo. 2017 Jan 28;8:2. doi: 10.1186/s13227-017-0065-y (PMC5273837; doi:10.1186/s13227-017-0065-y)
Supplement: Supplementary file 5 — Additional file 5. Spatial expression patterns of OSH1, LAX1, SPL14, miR156, miR529, LHS1, TAW1 and APO2 genes during panicle development of O. glaberrima and O. barthii. This figure illustrates the in situ hybridization data during panicle development not presented in Fig. 4 of the main text. [file 13227_2017_65_MOESM5_ESM.pdf]

OSH1

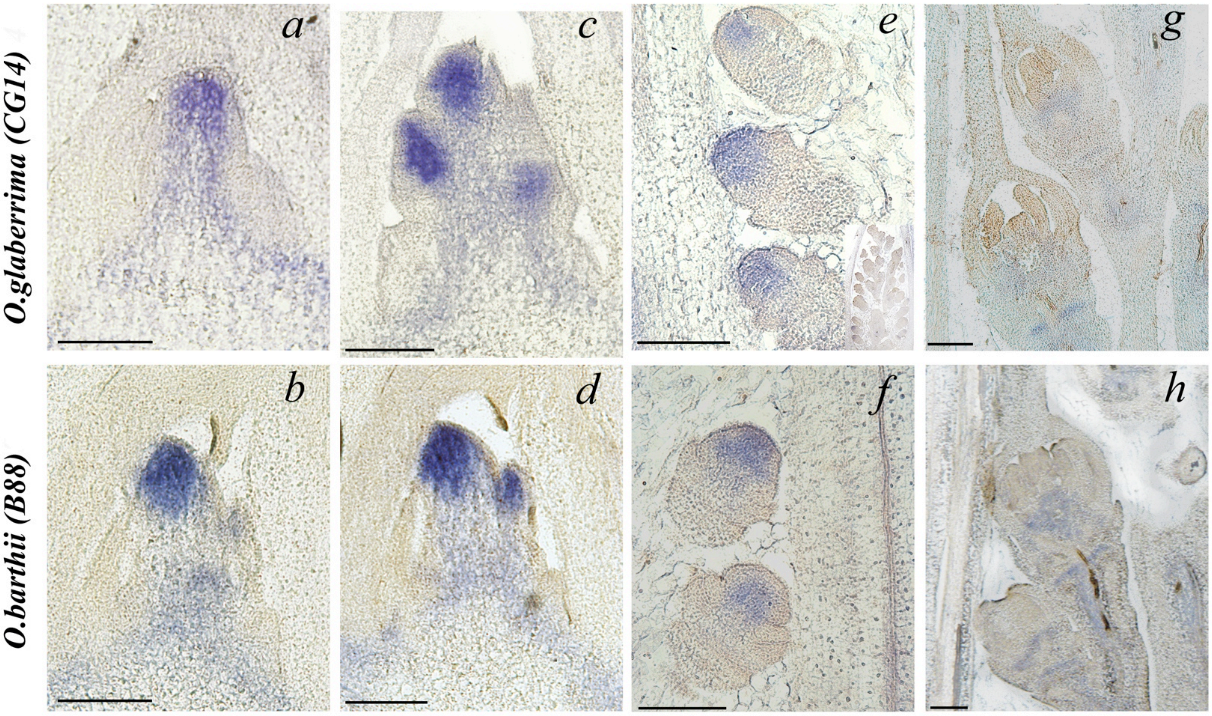

In situ mRNA patterns of *OSH1* gene at stages 1, 2 and 4 in *O. glaberrima* cv CG14 (a, c, e, g) and *O. barthii* var. B88 (b, d, f, h) respectively . Stage 1: a, b; stage 2: c, d, e, f; stage 4: g, h. Scale bars: 100µm.

LAX1

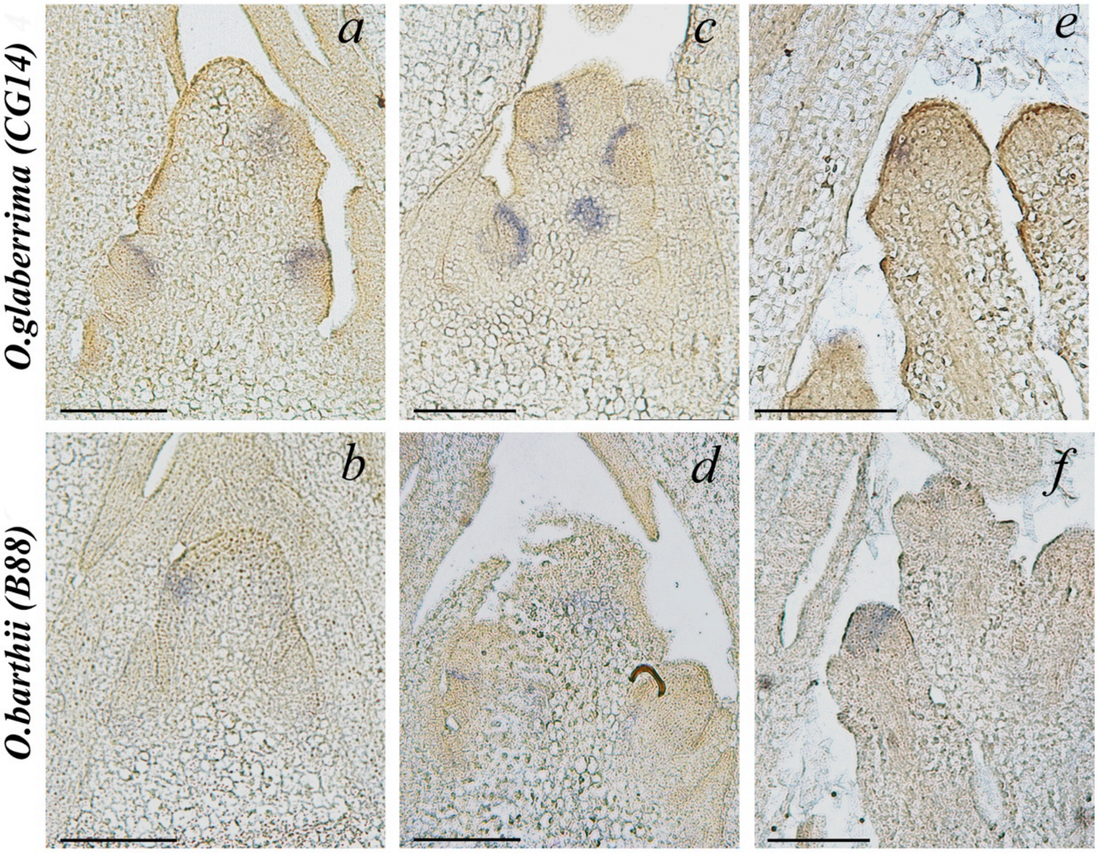

In situ mRNA patterns of *LAX1* gene at stages 1, 2 and 3 in *O. glaberrima* cv CG14 (a, c, e) and *O. barthii* var. B88 (b, d, f) respectively. Stage 1: a, b; stage 2: c, d; stage 3: e, f. Scale bars: 100µm.

SPL14

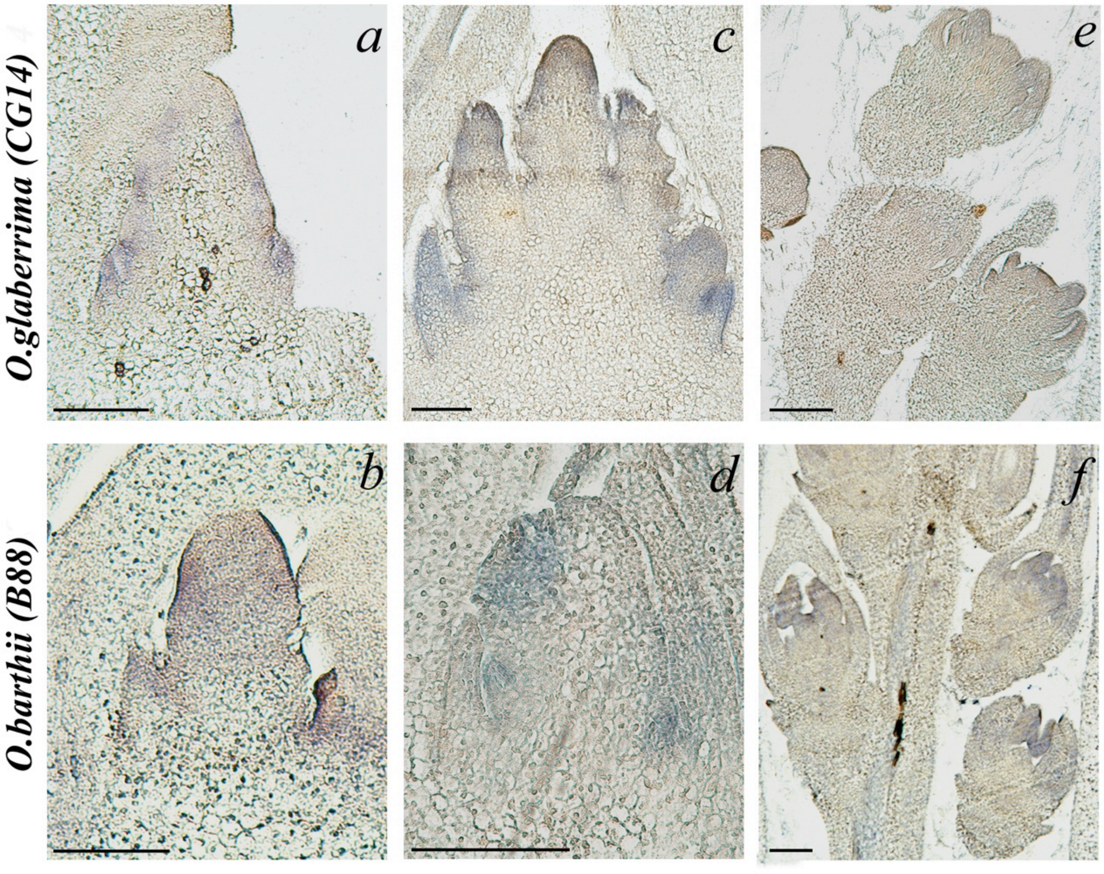

In situ mRNA patterns of *SPL14* gene at stages 1, 2 and 4 in *O. glaberrima* cv CG14 (a, c, e) and *O. barthii* var. B88 (b, d, f) respectively. Stage 1: a, b; stage 2: c, d; stage 4: e, f. Scale bars: 100µm.

miR156

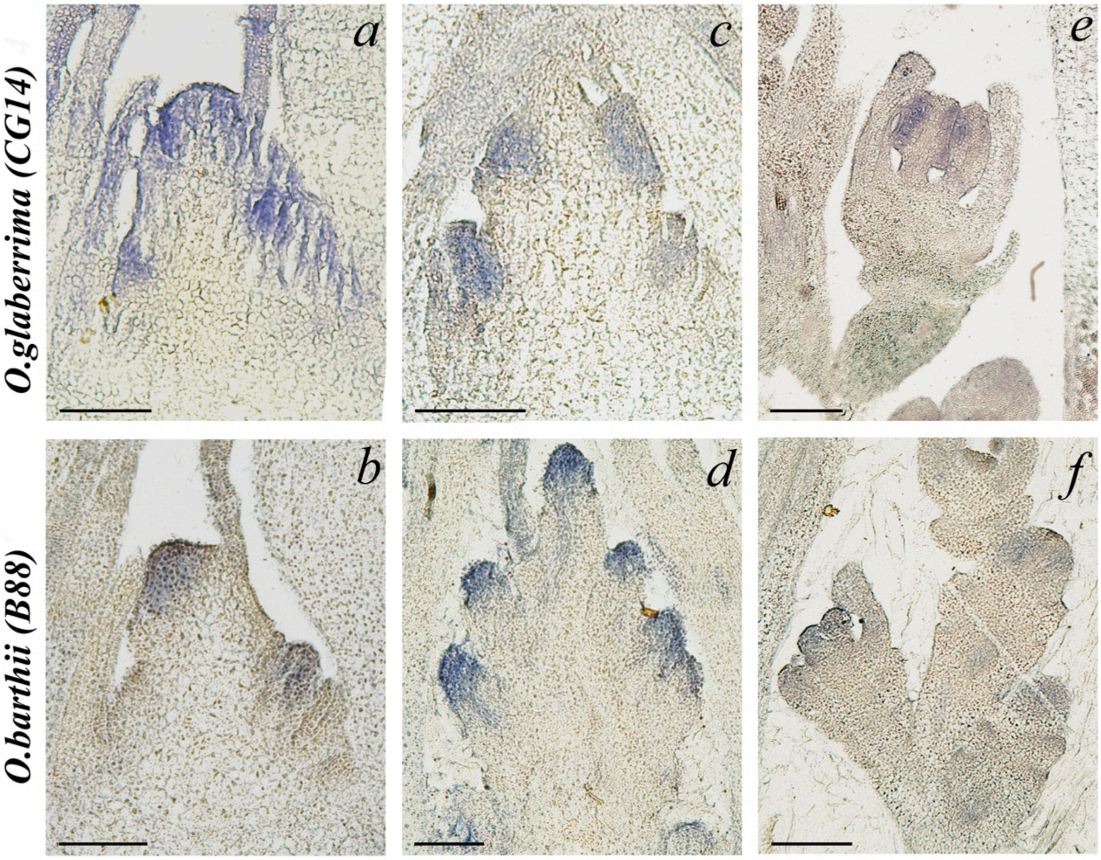

In situ mRNA patterns of *miR156* microRNA at stages 1, 2 and 4 in *O. glaberrima* cv CG14 (a, c, e) and *O. barthii* var. B88 (b, d, f) respectively. Stage 1: a, b; stage 2: c, d; stage 4: e, f. Scale bars: 100µm.

miR529

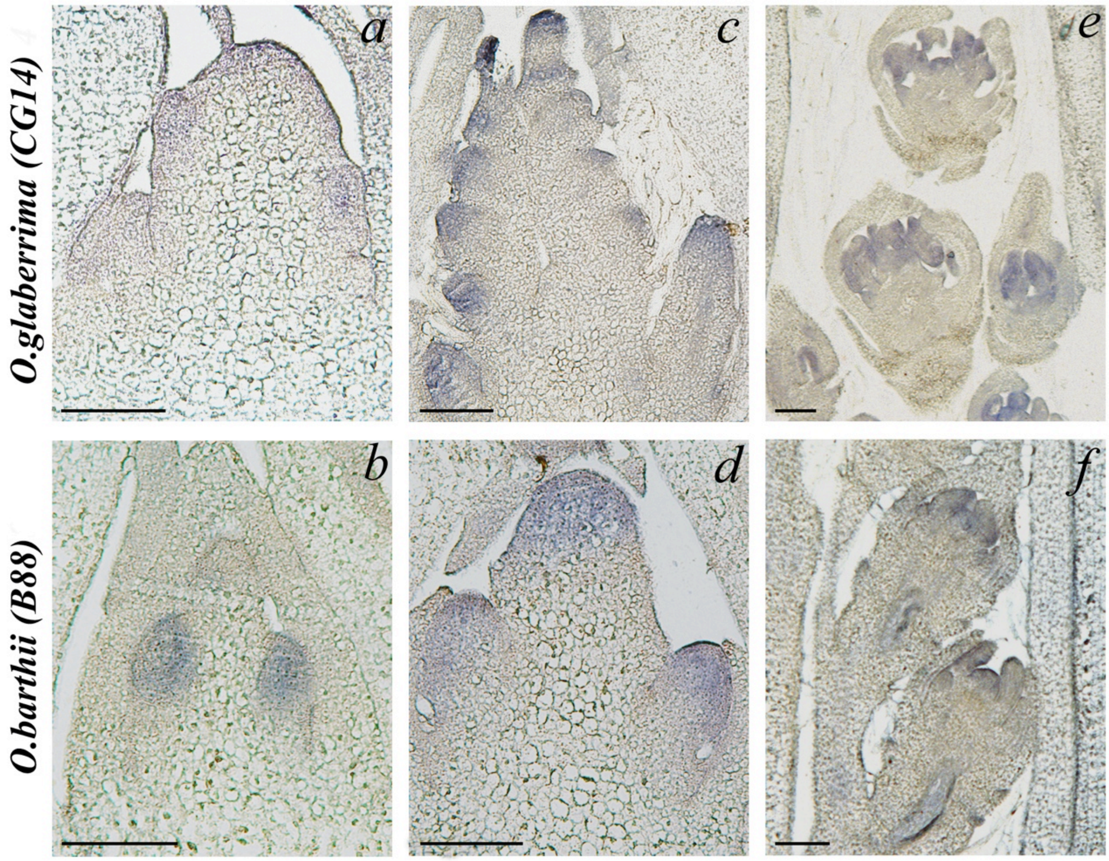

In situ mRNA patterns of *miR529* microRNA at stages 1, 2 and 4 in *O. glaberrima* cv CG14 (a, c, e) and *O. barthii* var. B88 (b, d, f) respectively. Stage 1: a, b; stage 2: c, d; stage 4: e, f. Scale bars: 100µm.

LHS1

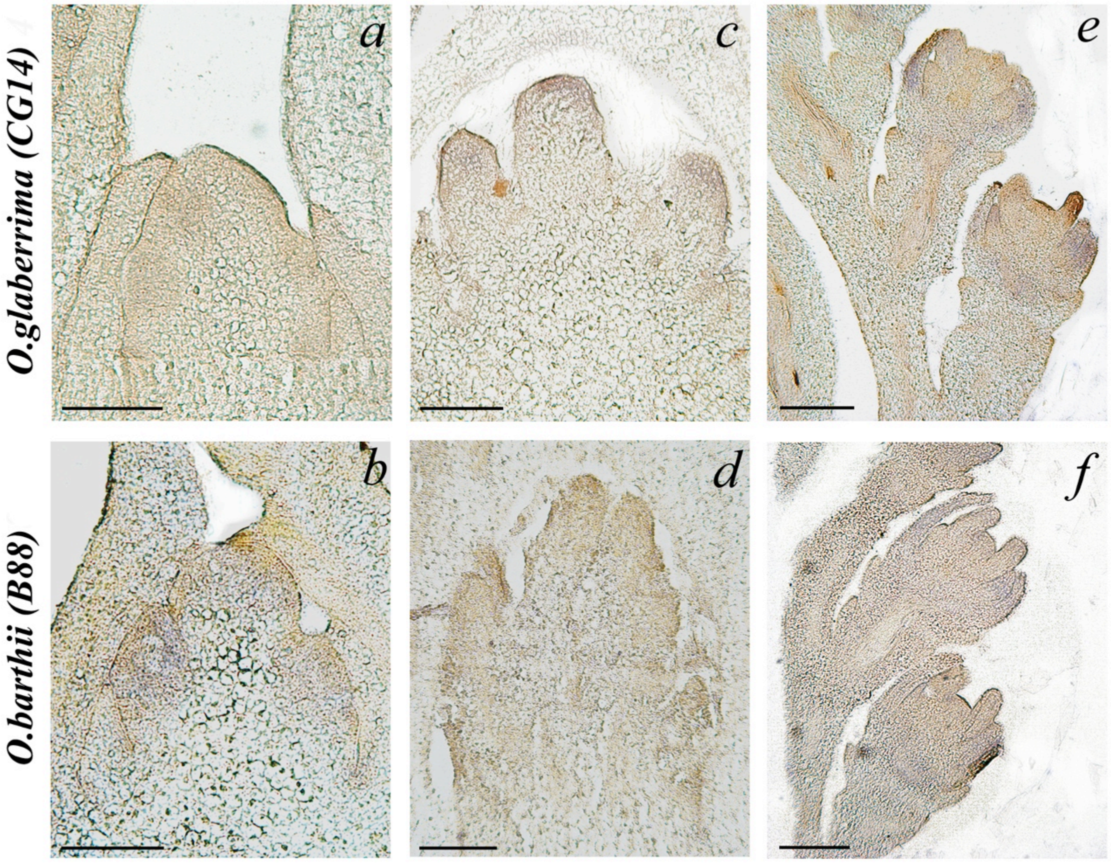

In situ mRNA patterns of *LHS1* gene at stages 1, 2 and 4 in *O. glaberrima* cv CG14 (a, c, e) and *O. barthii* var. B88 (b, d, f) respectively. Stage 1: a, b; stage 2: c, d; stage 4: e, f. Scale bars: 100μm.

APO2

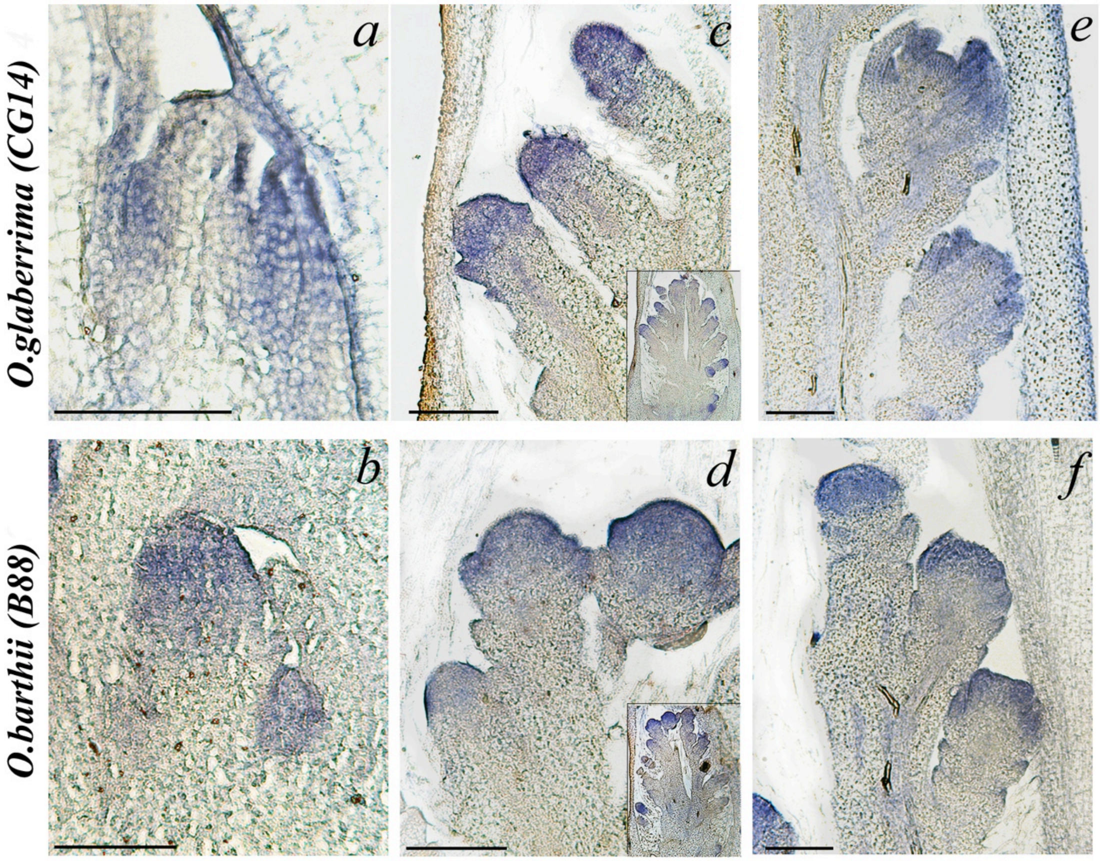

In situ mRNA patterns of *APO2* gene at stages 1, 3 and 4 in in *O. glaberrima* cv CG14 (a, c, e) and *O. barthii* var. B88 (b, d, f) respectively. Stage 1: a, b; stage 3: c, d; stage 4: e, f. Scale bars: 100µm.

TAW1

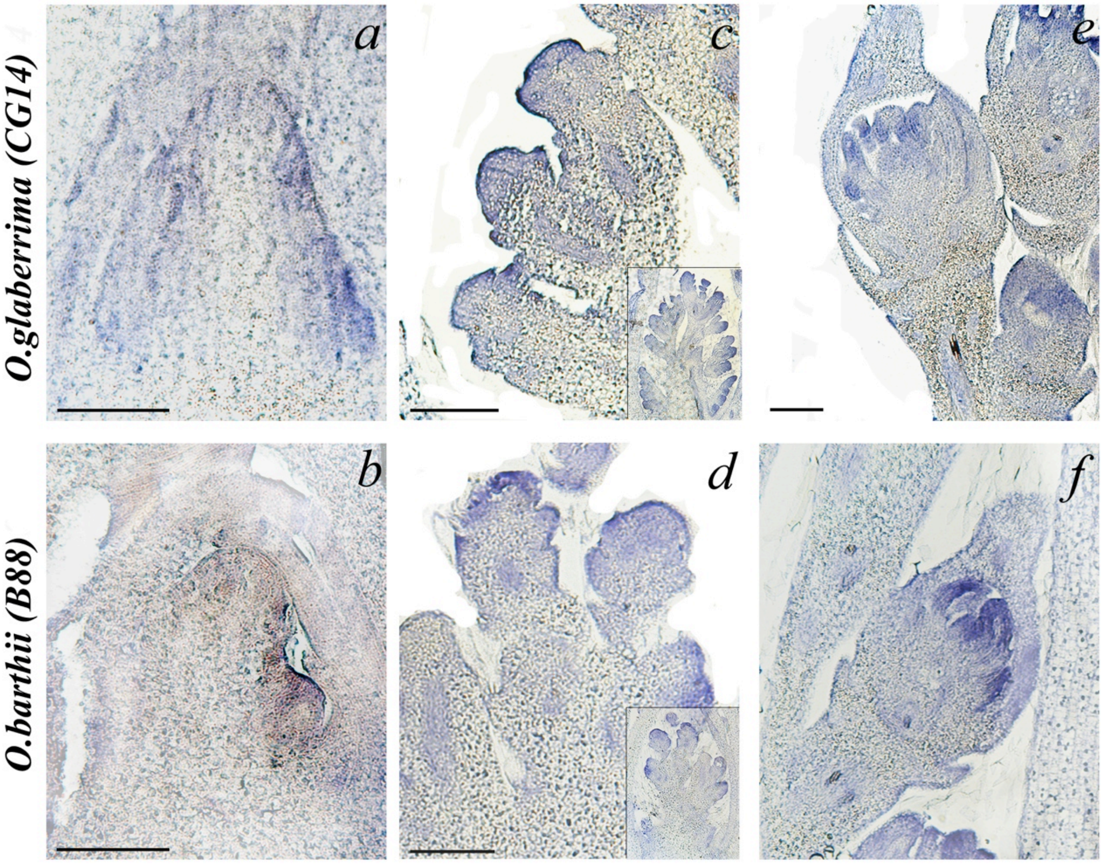

In situ mRNA patterns of *TAW1* gene at stages 1, 3 and 4 in in *O. glaberrima* cv CG14 (a, c, e) and *O. barthii* var. B88 (b, d, f) respectively. Stage 1: a, b; stage 3: c, d; stage 4: e, f. Scale bars: 100µm.
