# Supplementary material for: Differences in meristem size and expression of branching genes are associated with variation in panicle phenotype in wild and domesticated African rice
Source: EvoDevo. 2017 Jan 28;8:2. doi: 10.1186/s13227-017-0065-y (PMC5273837; doi:10.1186/s13227-017-0065-y)
Supplement: Supplementary file 7 — Additional file 7. miR156 and miR529 recognition sites in SPL14 mRNA target in O. sativa (OsSPL14), O. glaberrima (OgSPL14) and O. barthii (ObSPL14). The single nucleotide change in O. sativa from C to A at the Osa-miR156 targeted site in OsSPL14 as reported in the japonica cultivars Aikawa1 and Shaoniejing (SNJ) by [24, 25], respectively, is highlighted in red. Numbers above the sequence indicate the location of the nucleotide in the OsSPL14 coding sequence. Dots indicate identical nucleotide sequences in the region corresponding to the recognition sites of Osa-miR156 and Osa-miR529. [file 13227_2017_65_MOESM7_ESM.pdf]

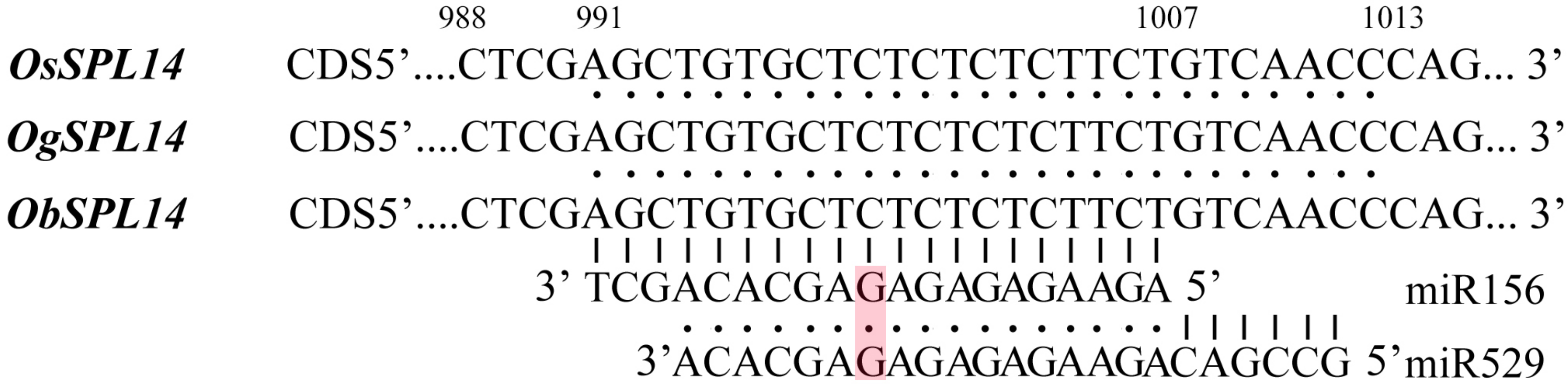
988
991
1007
1013
OsSPL14 CDS5’....CTCGAGCTGTGCTCTCTCTTCTGTCAACCCAG... 3’
OgSPL14 CDS5’....CTCGAGCTGTGCTCTCTCTTCTGTCAACCCAG... 3’
ObSPL14 CDS5’....CTCGAGCTGTGCTCTCTCTTCTGTCAACCCAG... 3’
3’ TCGACACGAGAGAGAGAAGA 5’ miR156
3’ACACGAGAGAGAGAAGACAGCCG 5’ miR529
