# Supplementary material for: Differences in meristem size and expression of branching genes are associated with variation in panicle phenotype in wild and domesticated African rice
Source: EvoDevo. 2017 Jan 28;8:2. doi: 10.1186/s13227-017-0065-y (PMC5273837; doi:10.1186/s13227-017-0065-y)
Supplement: Supplementary file 8 — Additional file 8. Promoter sequence comparisons of LHS1, APO2 and SPL14 orthologous genes in O. glaberrima and O. barthii. Vertical bars represent SNPs in O. barthii vs. O. glaberrima. Putative transcription factor binding sites related to SNPs are indicated by arrowheads (indicating site orientation). These binding sites are described as being involved in hormone response (ABA and jasmonate, JAS) [62, 63, 65] and in RNA polymerase II transcription activity [64]. The sequence alignments of these sites between O. barthii (Ob) and O. glaberrima (Og) are indicated on the right, and the corresponding polymorphic site is highlighted. [file 13227_2017_65_MOESM8_ESM.pdf]

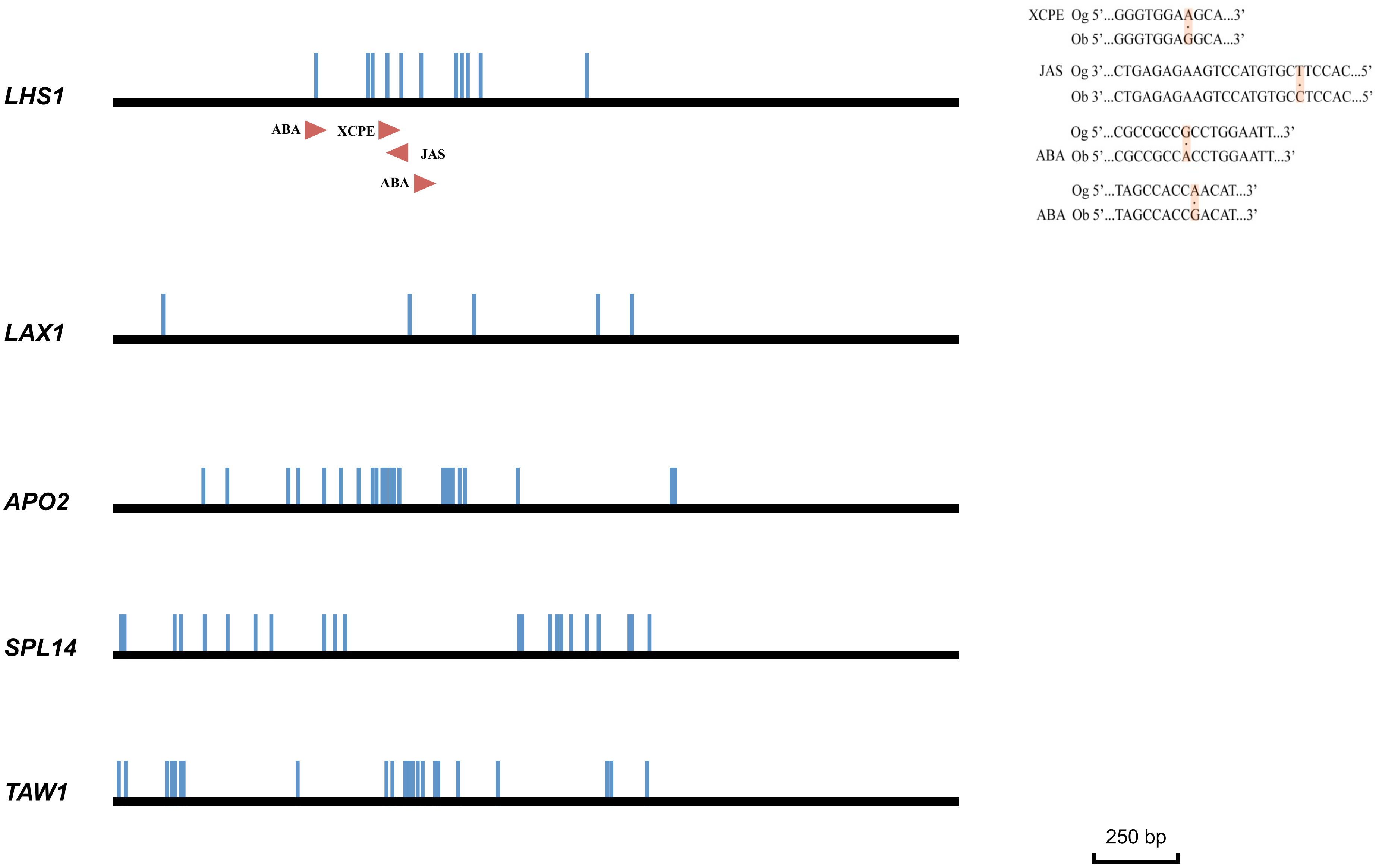
LHS1
ABA
XCPE
JAS
ABA
LAX1
APO2
SPL14
TAW1
XCPE Og 5'...GGGTGGAAGCA...3'
Ob 5'...GGGTGGAGGCA...3'
JAS Og 3'...CTGAGAGAAGTCCATGTGCTTCCAC...5'
Ob 3'...CTGAGAGAAGTCCATGTGCCTCCAC...5'
Og 5'...CGCCGCCGCCTGGAATT...3'
ABA Ob 5'...CGCCGCCACCTGGAATT...3'
Og 5'...TAGCCACCAACAT...3'
ABA Ob 5'...TAGCCACCGACAT...3'
250 bp
