# Supplementary material for: Differences in meristem size and expression of branching genes are associated with variation in panicle phenotype in wild and domesticated African rice
Source: EvoDevo. 2017 Jan 28;8:2. doi: 10.1186/s13227-017-0065-y (PMC5273837; doi:10.1186/s13227-017-0065-y)
Supplement: Supplementary file 9 — Additional file 9. List of primers used in this study. The underlined sequences correspond to the T7 promoter used for RNA probe synthesis. The sequences in italic correspond to the stem-loop region used for stem-loop qRT-PCRs. Bases shown in square brackets were LNA-modified for in situ hybridization. [file 13227_2017_65_MOESM9_ESM.pdf]

Additional File 9_Table S3: List of primers used in this study.

The underlined sequences correspond to the T7 promoter used for RNA probe synthesis. The sequences in italic correspond to stem-loop region for stem-loop qRT-PCRs. The base in square brackets were LNA-modified.

	Primer name	Sequence
**qRT-PCR**	ACT-F	CATTCCAGCAGATGTGGATTG
	ACT-R	TCTTGGCTTAGCATTCTTGG
	OSH1_F	CAGTTCGTGATGATGGAC
	OSH1_R	CTAAAACCGACCCCTGCATTA
	LAX1-F	ATTACCGGTTGGTCATGGTC
	LAX1-R	AAGCGATCGAGCAAACAAGT
	APO2-F	AGGTGCAATCCATGGCTAAG
	APO2-R	GCATCTTGGGCTTGTTGATG
	LHS1-F	GTGACCATTCCCTGCAGATT
	LHS1-R	GTCTGCTGCTTCATTGCTCA
	SPL14-F	CTGCCTGAATTTGACCAAGG
	SPL14-R	AAGCTTCTGAACCTGCGATG
	OsMADS22-F	CCCAAACCCAGACTGCAATA
	OsMADS22-R	CGAACAGTACAAGCGGAACA
	OsMADS55-F	CATCCACTCATGCACACAGA
	OsMADS55-R	GAGAGCTGAGAAATGGGATGA
	TAW1-F	CTAGTTACTCCACTCCACTC
	TAW1-R	GTAGTTTTGCTAGTAGCAAG
**Stem-loop qRT-PCR**		
	miR159b-RT	*GTCGTATCCAGTGCAGGGTCCGAGGTATTCGCACTGGATACGAC*CAGAGC
	miR159-F	CGGCGGTTTGGATTGAAGGGA
	miR529-RT	*GTCGTATCCAGTGCAGGGTCCGAGGTATTCGCACTGGATACGAC*AGGCTG

	miR529-F	CGGCGCAGAAGAGAGAGAGTA
	miR156-RT	*GTCGTATCCAGTGCAGGGTCCGAGGTATTCGCACTGGATACGAC*GTGCTC
	miR156-F	GCGGCGGTGACAGAAGAGAGT
	Univ-RT	GTGCAGGGTCCGAGGT
***in situ* hybridization**	OSH1_F	CAGTTCGTGATGATGGAC
	OSH1_R	CTAAAACCGACCCCTGCATTA
	OSH1_T7_F	GCGAAATTAATACGACTCACTATAGGGCGAACAGTTCGTGATGATGGAC
	OSH1_T7_R	GCGAAATTAATACGACTCACTATAGGGCGAACTAAAACCGACCCCTGCATTA
	LAX1_F	CTTCGTCCAGGAGACTGACC
	LAX1_R	TTAAGGGACCATGACCAACC
	LAX1_T7_F	GCGAAATTAATACGACTCACTATAGGGCGAACTTCGTCCAGGAGACTGACC
	LAX1_T7_R	GCGAAATTAATACGACTCACTATAGGGCGAATTAAGGGACCATGACCAACC
	OsSPL14_HIS_F	AGTGGCACAGGAACGTAGCTCCT
	OsSPL14_HIS_R	GCACAGCTCGAGTCGGTGGCGGCAC
	OsSPL14_T7_HIS_F	GCGAAATTAATACGACTCACTATAGGGCGAAAGTGGCACAGGAACGTAGCTCCT
	OsSPL14_T7_HIS_R	GCGAAATTAATACGACTCACTATAGGGCGAAGCACAGCTCGAGTCGGTGGCGGCAC
	APO2_F	ATCTCGGAGCTCGGGTTCACG
	APO2_F1	GCCGACCGCAAGGACAGCAA
	APO2_R	CGCAAACATGGGTACACGACG
	APO2_T7F1	GCGAAATTAATACGACTCACTATAGGGCGAA GCCGACCGCAAGGACAGCAA
	APO2_T7R	GCGAAATTAATACGACTCACTATAGGGCGAA CGCAAACATGGGTACACGACG
	LHS1-HIS-F	GAAGAGCAAGGAGCAACAGC
	LHS1-HIS-R	AATCTGCAGGGAATGGTCAC
	LHS1-HIS-T7F	GCGAAATTAATACGACTCACTATAGGGCGAAGAAGAGCAAGGAGCAACAGC
	LHS1-HIS-T7R	GCGAAATTAATACGACTCACTATAGGGCGAAAATCTGCAGGGAATGGTCAC
	TAW1_insitu_F	GCGTCAGCTACGAGAAGAAG
	TAW1_insitu_R	GTAGTTTTGCTAGTAGCAAG

	TAW1_insitu_T7+F	GCGAAATTAATACGACTCACTATAGGGCGAAGCGTCAGCTACGAGAAGAAG
	TAW1_insitu_T7+R	GCGAAATTAATACGACTCACTATAGGGCGAAGTAGTTTTGCTAGTAGCAAG
	T7_insitu	GCGAAATTAATACGACTCACTATAGGGCGAA
	LNA-miR156 (Exiqon)	GTGCTCACTCTCTTCTGTCA
	LNA-miR529	AGGC[G]TAC[C]TCTC[C]T[C]T
**Sequencing**	APO2_SEQ_OB_F2	ATCCCAACGATGCCTTCTCGG
	APO2_SEQ_F1	CGCAAGGACAGCAAGCTAGTA
	APO2_SEQ_R1	GCTCCCCGCCATGTCATGCTC
	SPL14_SEQ_OB_F1	GGAGAGAAAGGAGGCTCGTCGG
	SPL14_SEQ_OB_F2	CACTGTGGGTGCAGTGTCTT
	SPL14_SEQ_OB_R1	AGCAAAGCAAAAGCAGTGGT
